# Semi-automated identification of biological control agent using artificial intelligence

**DOI:** 10.1038/s41598-020-71798-x

**Published:** 2020-09-03

**Authors:** Jhih-Rong Liao, Hsiao-Chin Lee, Ming-Chih Chiu, Chiun-Cheng Ko

**Affiliations:** 1 Department of Entomology, National Taiwan University, Taipei City, 10617 Taiwan; 2 Institute of Hydrobiology, Chinese Academy of Sciences, Wuhan City, 430072 China

**Keywords:** Biological techniques, Ecology, Zoology

## Abstract

The accurate identification of biological control agents is necessary for monitoring and preventing contamination in integrated pest management (IPM); however, this is difficult for non-taxonomists to achieve in the field. Many machine learning techniques have been developed for multiple applications (e.g., identification of biological organisms). Some phytoseiids are biological control agents for small pests, such as *Neoseiulus barkeri* Hughes. To identify a precise biological control agent, a boosting machine learning classification, namely eXtreme Gradient Boosting (XGBoost), was introduced in this study for the semi-automated identification of phytoseiid mites. XGBoost analyses were based on 22 quantitative morphological features among 512 specimens of *N. barkeri* and related phytoseiid species. These features were extracted manually from photomicrograph of mites and included dorsal and ventrianal shield lengths, setal lengths, and length and width of spermatheca. The results revealed 100% accuracy rating, and seta *j4* achieved significant discrimination among specimens. The present study provides a path through which skills and experiences can be transferred between experts and non-experts. This can serve as a foundation for future studies on the automated identification of biological control agents for IPM.

## Introduction

Integrated pest management (IPM) is applied in agricultural practices to concurrently minimise crop damage, environmental contamination, and economic loss^1,2^. Biological control agents are crucial for IPM and agroecosystems. Regularly monitoring pests and biological control agents is necessary for IPM. Without accurate identification, the efficiency of biological control cannot be monitored^2,3^. In addition, companies currently breed biological control agents at large scales for commercial purposes. Contamination by various biological control agent species may become a serious problem because of their highly specialised functions^4^.

Machine learning is a form of artificial intelligence in which a computer learns from previous examples (called training) and subsequently performs task using new data (called inference)^5^. Morphological identification requires expert knowledge, and machine learning, such as through the use of eXtreme Gradient Boosting (XGBoost), can help boost scientific development. The use of fully verified and professional specimen-making techniques (e.g., the slide-making technique for mites) and a detailed understanding of structures are required. For example, distinguishing feature states (e.g., longer or shorter setae) are difficult to identify without experience^6^. The application of statistical methods to distinguish morphometric features may be instrumental to the reliable discrimination of species^6–9^. The automated identification of biological organisms has been a goal for centuries^10–12^. This may gradually be achieved through the many machine learning techniques that have been developed since the rapid evolution of computation began. Deep learning (DL) is the most well-known technique in multiple domains and is particularly suitable for automatically identifying images^3,13,14^. For example, Fedor et al.^14^ identified 18 thrip species by using an artificial neural network and achieved 97% reliability. However, DL has the following limitations in identifying biological organisms: (1) large datasets are required, (2) labelling errors can occur, even among experts, (3) DL models can learn to solve only some problems particularly well, (4) it is time consuming, and (5) available datasets do not completely describe the problems they target^13^. Moreover, Gaston and O’Neill^10^ reviewed the difficulties of automated identification, which include (1) variations in morphological features from various causes, (2) the difficulty of capturing critical detailed features, and (3) the enormous number of described and unknown species. Missing data is also a problem. XGBoost has received attention because it is highly effective, saves considerable time^15,16^, and provides solutions for these problems. It can be applied to supervised learning tasks, such as regression and classification. Several advantages of XGBoost have been reported, including its rapid computational speed, favourable model performance, ability to manage sparse or missing data, and low specimen requirements^16–18^. Identification using XGBoost has recently become popular in studying medicine^18^ and plant diseases^19^. Therefore, these characteristics of XGBoost make it suitable for use on biological organisms.

The mite family Phytoseiidae (Acari: Mesostigmata) inhabits diverse habitats, such as agronomic crops, horticultural crops, xylophyta, herbaceous plants, and weeds^20,21^. Some members of the family are predators of phytophagous mites and other small arthropods, whereas others feed on fungal spores, leaf juices, pollen grains, soil litter, and other plant materials^21–23^. They have received much attention because of their potential as biological control agents in IPM^21^. The predatory mite *Neoseiulus barkeri* Hughes is a species found worldwide and distributed across Asia, the Americas, Australia, Africa, and Europe^24,25^; it can be used as a biological control agent. McMurtry et al.^21^ proposed a lifestyle classification of phytoseiid mites, which included four types and ten subtypes. *N. barkeri* is a generalist predator with a type III lifestyle, meaning that it feeds on mite pests, some small insects such as thrips and whiteflies, and various pollens^21,26–31^. This species is also known for controlling *Thrips tabaci*^26,29^, *Polyphagotarsonemus latus*^27^, and *Tetranychus urticae*^30,31^.

To help non-taxonomists identify the aforementioned biological control agents, a semi-automated identification method using XGBoost based on morphological features (e.g., dorsal setal length) was developed. The dorsal setal lengths of phytoseiid mites have long been studied to identify them and reconstruct their phylogeny^7,8,33,34^. In the present study, taxonomists first selected morphological characteristics. Subsequently, we manually extracted quantitative measurements for analysis. Therefore, the method in this study was semi-automated. Automatic identification by using photomicrograph to study mites is difficult because of their small body size and the complexity of phytoseiid morphology. This is also time-consuming because microscopic photos must be used. Furthermore, dorsal setae are occasionally missing because of the slide-making procedure. XGBoost was advantageous in the present study for its rapid computational speed and ability to function even with missing data. Therefore, we developed a method for the semi‐automatic identification of *N. barkeri* and related species on the basis of their morphological features*.*

## Results

**Figure 1:**
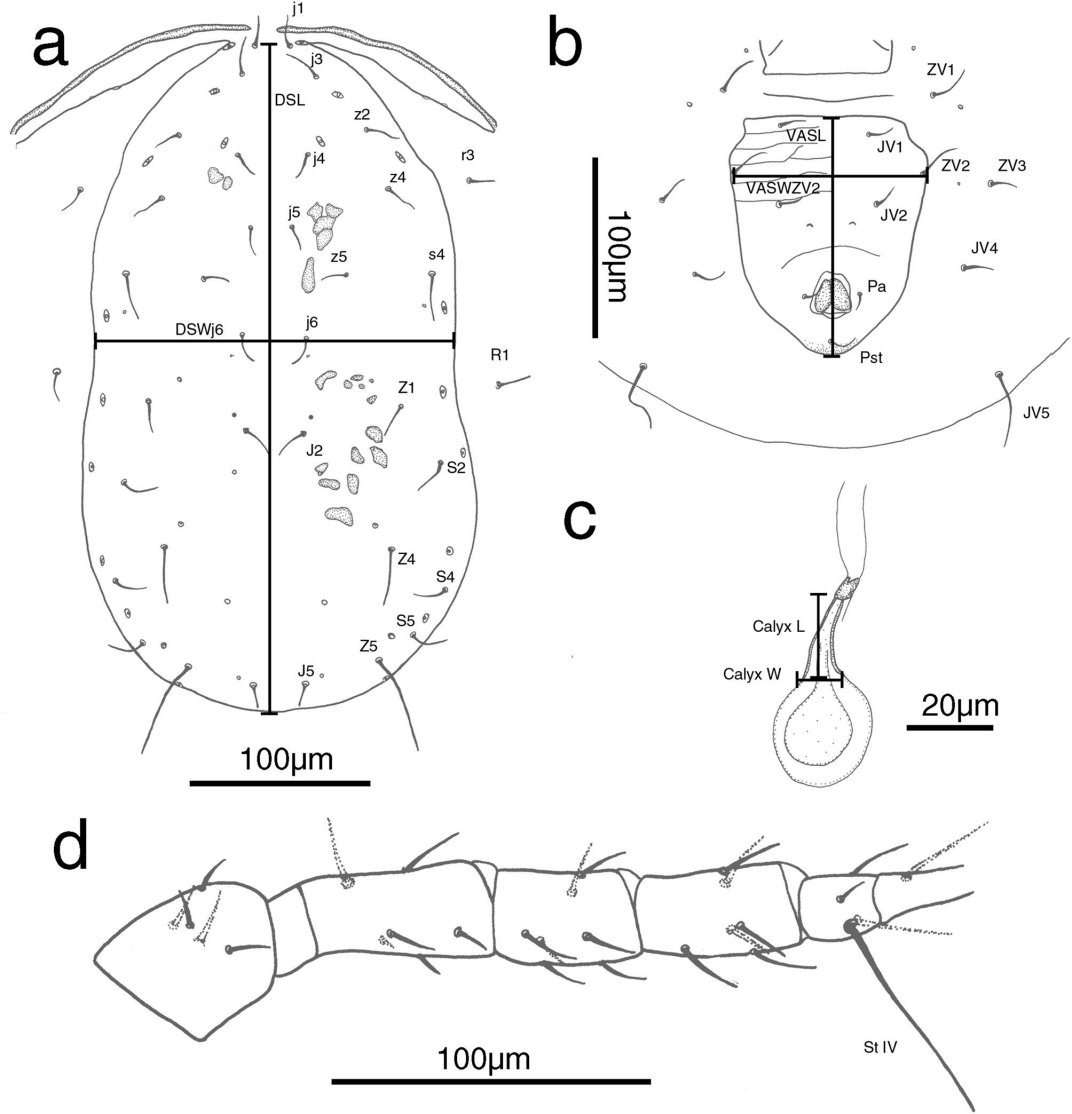
Measured variables of female phytoseiid mites: (**a**) dorsal shield, (**b**) ventral view, (**c**) spermatheca, (**d**) leg IV. Dorsal aspect: DSL, dorsal shield length; DSWj6, dorsal shield width at seta *j6* level. Ventral aspect: VASL, ventrianal shield length; VASWZV2, ventrianal shield width at seta *ZV2* level. Spermatheca aspect: Calyx L, spermatheca calyx length; Calyx W, spermatheca calyx width. Leg aspect: *St* IV, macroseta on barsitarsus IV.

We collected and captured microscopic images of 512 collected female phytoseiid specimens. Subsequently, from the microscopic images, we manually recorded 22 morphological measurements, including the lengths and widths of dorsal and ventrianal shields, lengths of 14 pairs of dorsal setae, length of ventral seta *JV5*, length of macroseta *St* IV, and length and width of the spermatheca calyx (Fig. 1, Table 1, Table S1).

**Table 1:** List and abbreviations of morphological features (μm).

	Abbreviation	Morphological features
1	DSL	Dorsal shield length
2	DSW *j6*	Dorsal shield width at *j6* level
3	*j1*	Length of the seta *j1*
4	*j3*	Length of the seta *j3*
5	*j4*	Length of the seta *j4*
6	*j6*	Length of the seta *j6*
7	*J5*	Length of the seta *J5*
8	*z2*	Length of the seta *z2*
9	*z4*	Length of the seta *z4*
10	*z5*	Length of the seta *z5*
11	*Z1*	Length of the seta *Z1*
12	*Z4*	Length of the seta *Z4*
13	*Z5*	Length of the seta *Z5*
14	*s4*	Length of the seta *s4*
15	*r3*	Length of the seta *r3*
16	*R1*	Length of the seta *R1*
17	VSL	Length of the ventrianal shield
18	VSW	Width of the ventrianal shield (at *ZV2* level)
19	*JV5*	Length of the seta *JV5*
20	*St* IV	Length of the macroseta *St* IV on the basitarsus of leg IV
21	Calyx L	Length of the calyx of spermatheca (without atrium)
22	Calyx W	Width of the calyx of spermatheca (at widest level)

**Figure 2:**
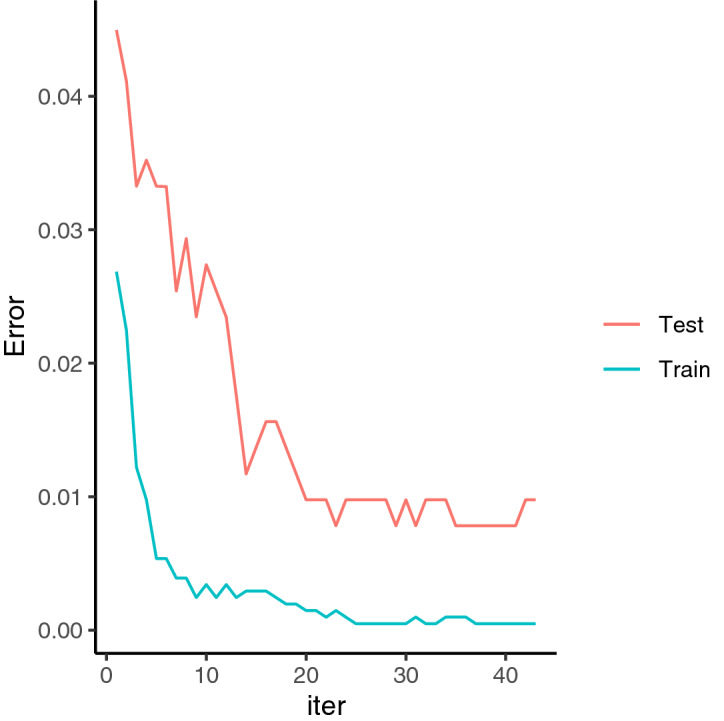
Classification error of modelling with training data (blue line) and cross-validation test (red line) along with the number of trees (iter). The binary classification error rate was calculated as the number of incorrect case divided by the total number of all cases.

During cross-validation, the number of errors in distinguishing the target species (i.e., *N. barkeri*) decreased as the number of decision trees increased and the XGBoost model fitted to training dataset. By contrast, the number of prediction errors of the fitted model regarding the morphological features of the testing dataset was lower with a greater number of trees but was generally higher than of the model during the training process. Through cross-validation and after 23 decision trees has been generated, the training and testing processes attained the same performance level in XGBoost modelling, and they achieved the identification accuracy of 100 and 99%, respectively (Fig. 2). Finally, with all datasets and the determined number of decision trees, the identification accuracy reached 100%.

**Figure 3:**
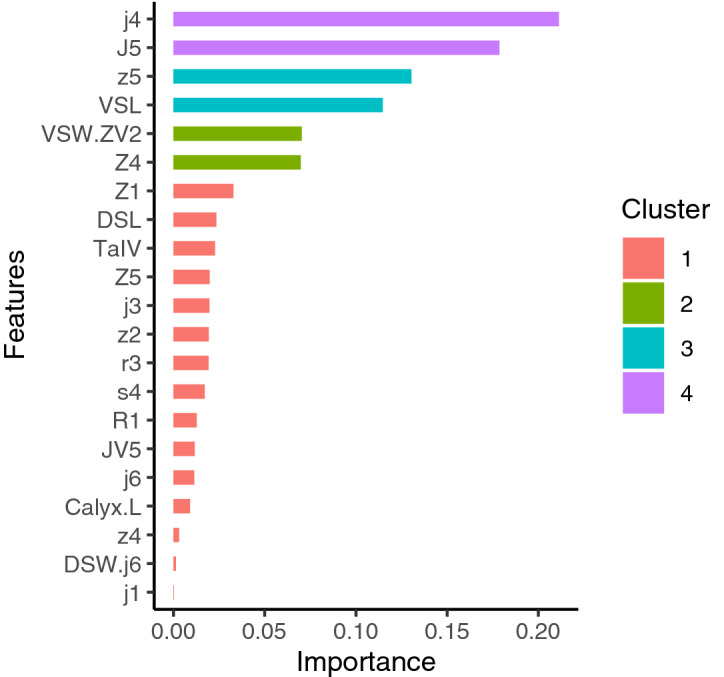
Relative importance of features, with automatically divided clusters in the eXtreme Gradient Boosting model.

**Figure 4:**
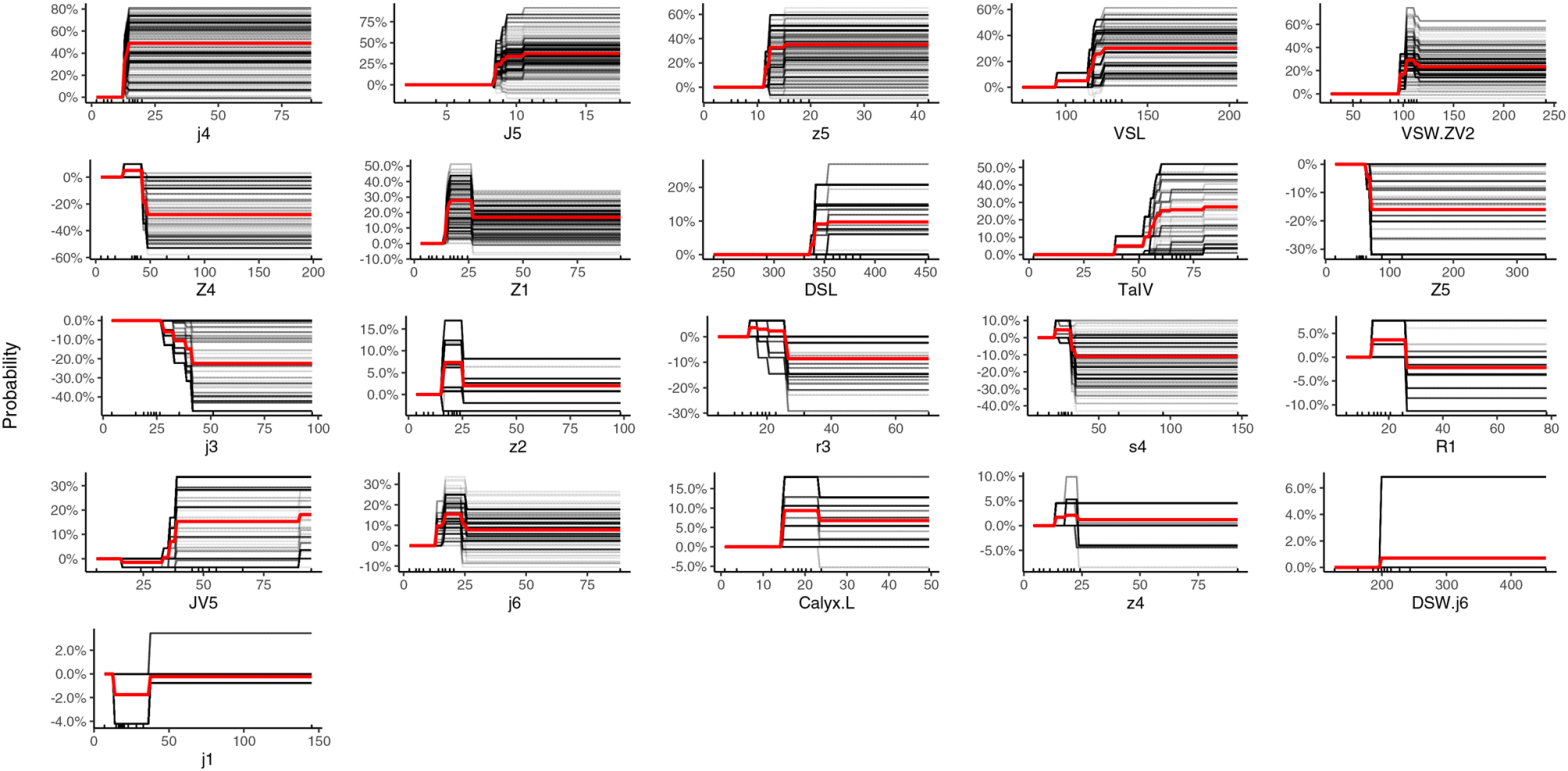
Centred individual conditional expectation plot for predicted probability of being the target species (*Neoseiulus barkeri*) based on 21 key features (μm). The lines reveal the difference in prediction from that with the respective feature value at its observed minimum.

Because the number of decision trees was determined through cross-validation, the results of the final model using full data exhibited zero errors in distinguishing between the target species and related species. In addition, this final model indicated key morphological features (according to their importance; Fig. 3), and the individual conditional expectation (ICE) plots exhibited their determinative roles (Fig. 4). Twenty-one features (not all) were considered critical for target species identification. Furthermore, these key features were assigned to four clusters with different levels of importance. Their determinative roles influenced the probability of the target species having several patterns (unimodal, monotonically increasing or decreasing, or more complex patterns). For example, seta *j4* was the most crucial feature (more than 40%) and exhibited a unimodal pattern. The predicted probability of being the target species was clearly higher when the length of seta *j4* varied within the range of and 30 μm. In addition, this probability decreased for other higher or lower values of seta *j4* relative to that for the middle range. By contrast, the seta *J5* was the second most crucial feature. Rather than exhibiting a unimodal pattern, a longer length of seta *J5* monotonically increased with an increase in the probability of being the target species. A monotonically decreasing pattern was identified for the length of seta *j3*, which was considered the 11th most crucial feature. Notably, the spermatheca was the most crucial identifying feature, but it was ranked 18th in terms of feature importance (Figs. 3 and 4).

## Discussion

To the best of our knowledge, the present study is the first to use a machine learning technique to identify biological control agents for agricultural use and is also the first such study on mites. The results achieved 100% accuracy in distinguishing between target species and related species. The high percentage of correctly identified specimens from our data set suggests that the practical use of XGBoost for phytoseiid identification may become more prevalent. Generally, more predictors (i.e., features in our study) for a certain number of samples (i.e., specimens) can improve modelling performance. Although overfitting occurs when noise is mistaken for information, validation helps to prevent this problem from influencing model performance. After validation, high accuracy (100%) was retained. This was unsurprising because a taxonomist selected morphological traits that have long been considered useful for identification.

### Drawbacks of machine learning techniques

The major drawbacks of DL include the considerable data and time required to achieve adequate accuracy^13^. Furthermore, such an approach cannot be applied in every situation (e.g., the identification in our study). Nevertheless, we resolved the problems of insufficient and missing data by using machine learning to identify biological organisms. XGBoost saves considerable time and achieves more reliable identification than does DL (e.g., Fedor et al.^14^). This method is convenient for non-experts to use; however, several limitations must be mentioned. First, DL can only import raw image data or extract features automatically. However, features must be extracted first when XGBoost is applied; therefore, the selection of morphological features in the first step is crucial. Second, only the taxa included in the training set can be identified using the XGBoost algorithm. The identification of novel and excluded species is problematic for XGBoost and all machine learning techniques^10^. Third, the morphological boundaries of closely related species may not be recognised through machine learning identification. This is a common problem in identification of biological organisms.

### Key morphological features and their roles

XGBoost could detect interspecific variations among phytoseiid mites in our study. We collected *N. barkeri* specimens from various countries, including the United States, China, Israel, Japan, the Netherlands, Taiwan, and Thailand. All specimens were identified accurately. This result indicated that XGBoost can classify target species with intraspecific variations among phytoseiid mite specimens from around the world. For comparison with our target species *N. barkeri*, 35 non-target speciesof the subfamily Amblyseiinae were selected. Dorsal setae *j2*, *j5*, *J2*, *S2*, *S4*, and *S5* were excluded from the comparison. The present results indicated that XGBoost can overcome interspecific variations among phytoseiid mite specimens. However, for the classification of more taxa, setal patterns may present a limitation for comparison, and more morphological features should be chosen.

Some features are crucial for mite identification through machine learning. The results presented here reveal that 22 quantitative features can be helpful for identifying phytoseiid mites. Most of these measurements involved setal length, and the results indicated that seta *j4* is one of the most critical features. Dorsal setae are the most critical features for phytoseiid identification and phylogeny^32^. However, the morphological features have intraspecific variations within species caused by factors such as gene expression, life stage, and environmental conditions^10^. Variations in setal length and pattern can occur in phytoseiid mites^8,34^. Tixier et al.^7^ reported significant differences in setal lengths between winter and summer seasons, indicating that additional features are necessary for species identification. Tixier^8^ reported on setal length variations in phytoseiid mites and recommended using at least 10 specimens for comparison. Moreover, apart from aspects related to the mites themselves, two possible reasons for errors should be considered: measurement errors^5^ reveal a need for standard measurement among experts; and excessive use of medium while making slides influences setal length. The slide-making technique also affects the observation of morphological features^6^, such as chelicera dentition and spermatheca shape, both of which are key identifying features. However, Toyoshima and Amano^34^ reported six conditions of setal pattern variation in phytoseiid mites: (1) absent seta, (2) additional seta, (3) inserted seta, (4) deviated seta, (5) expanded seta, and (6) shortened seta. They considered such phenomena to be caused not by accidents during post-embryonic development but by heritable features. Future efforts can be focused on the association between phylogeny and ML classification; this association may provide a basis for mechanistic explorations.

## Conclusions

The automated identification of biological organisms may gradually become a reality through the development of machine learning techniques, which are widely applied in domains such as medicine^18^ and agriculture^14,19^. The automated extraction of morphological features is a method that will be used in the future but requires more development in the context of mite research. Many studies have reported on the automatic extraction of morphological features, such as insect wing veins^3,9^; however, mite studies have many limitations. High-quality slide specimens and high-resolution microscopic photos are required. Porto and Voje^9^ proposed a machine learning pipeline for automated detection and landmarking of biological structures in images. This method may enable the collection of morphological data from images. However, the main morphological features of phytoseiid mites are setal patterns (presence or absence) and setal lengths^8,32^. Detecting setae is impossible because their direction of setae may vary and can be affected by the slide-making technique. In addition, Prasad and Tixier^33^ indicated that the difficulty in obtaining accurate length measurements is due to setae not being straight and having thin and pointed tips. To develop automated identification techniques for phytoseiid mites, additional features must be used for identification, such as the distances between setae, distance between pores, and shield width and length. However, tabletop scanning electronic microscopes (TSEMs) may be another method for capturing high-resolution photos. TSEMs have recently begun being applied in mite studies; for example, Yamasaki et al.^35^ compared light microscope and TSEM photos of feather mites. TSEMs have several advantages, such as the ability to capture the living positions of specimens, simple operability by non-experts, and no need for pre-processing.

Our study is the first to successfully identify biological control agents through artificial intelligence. Accurately identifying pests is the first step of IPM (e.g., Fedor et al.^14^); however, the accurate identification of biological control agents is also necessary^2^. The present study developed a semi-automated identification method for phytoseiid mites by using XGBoost, which can facilitate the accurate identification of target and non-target phytoseiid species for IPM. After morphological features of phytoseiid mites were selected in accordance with experts’ recommendations, the machine learning technique could bridge the gap between taxonomists and non-taxonomists, through the transfer of taxonomy skills and experience. This approach can also provide a quick method for dealing with insufficient or missing data. Therefore, XGBoost can be used for the identification of mites and for that of all similar animals in which morphometrics are used extensively, such as aphids and whiteflies. We consider the present study to be the first step toward realising mite identification through artificial intelligence. Based on the application potential, more species and individuals that more possibly provides the entire ranges and variations of morphometric features before XGBoost identification can become more reliable in all situations. The practical application of artificial intelligence is expected to overcome all identification problems for IPM in the future.

## Methods

### Sampling of *N. barkeri* and related species

Phytoseiid mites inhabit a variety of habitats, such as various plants and soil litters. Individuals were collected from plants and those on substrates and soil litters were isolated using Berlese’ funnels and kept in 95% alcohol. Samples were mounted in Hoyer’s medium and softened and cleaned with lactic acid if the mite body was hard. In addition, specimens were deposited at several institutes: GIABR (Guangdong Institute of Applied Biological Resources, Guangzhou, Guangdong, China), HUM (Hokkaido University Museum, Sapporo, Japan), NMNS (National Museum of Nature and Science, Tsukuba, Japan), NTU (Department of Entomology, National Taiwan University, Taipei, Taiwan), TARL (Taiwan Acari Research Laboratory, Taichung City, Taiwan). Female phytoseiid mites were collected, including 250 specimens of *N. barkeri*, and 262 specimens of 35 non-target species belonging to subfamily Amblyseiinae, in 6 tribes, and 11 genera. The following numbers of these non-target species were collected: 4 of *N. baraki*, 10 of *N. longispinosus*, 10 of *N. makuwa*, 6 of *N. taiwanicus*, 9 of *N. womersleyi*, 9 of *Amblyseius alpinia*, 10 of *A. bellatulus*, 10 of *A. eharai*, 10 of *A. herbicolus*, 2 of *A. pascalis*, 10 of *A. tamatavensis*, 10 of *Euseius aizawai*, 6 of *E. circellatus*, 7 of *E. daluensis*, 11 of *E. macaranga*, 10 of *E. ovalis*, 6 of *E. paraovalis*, 3 of *E. nicholsi*, 6 of *E. oolong*, 7 of *E. sojaensis*, 4 of *Gynaeseius liturivorus*, 3 of *G. santosoi*, 10 of *Okiseius subtropicus*, 4 of *Paraamblyseius formosanus*, 7 of *Paraphytoseius chihpenensis*, 10 of *Parap. cracentis*, 3 of *Parap. hualienensis*, 10 of *Parap. orientalis*, 6 of *Phytoscutus salebrosus*, 10 of *Proprioseiopsis asetus*, 3 of *Prop. ovatus*, 8 of *Scapulaseius anuwati*, 10 of *S. cantonensis*, 10 of *S. okinawanus*, and 8 of *S. tienhsainensis*. In addition, specimens of *N. barkeri* were collected from the United States, China, Israel, Japan, the Netherlands, Taiwan, and Thailand (including intercepted specimens in plant quarantine).

### Quantitative measurements of phytoseiid mites

Specimens were examined under an Olympus BX51 microscope, and measurements were performed using a stage-calibrated ocular micrometer and ImageJ 1.47^36^. Photos were taken using a Motic Moticam 5+ camera attached to the microscope (Figure S1). All measurements were recorded in micrometres (μm). The general terminology used for morphological descriptions in this study conformed to that of Chant and McMurtry^20^. The notation for idiosomal setae conformed to that of Lindquist and Evans^37^ and Lindquist^38^, as adapted by Rowell et al.^39^ and Chant and Yoshida-Shaul^32^. Phytoseiid mites exhibit pronounced sexual dimorphism, and female individuals are more crucial for identification because of their distinguishing features and greater prevalence. In the present study, 22 quantitative measurements were collected from the female specimens: dorsal shield length and width; *j1*, *j3*, *j4*, *j6*, *J5*, *z2*, *z4*, *z5*, *Z1*, *Z4*, *Z5*, *s4*, *r3*, and *R1* setae length; ventrianal shield length and width (at *ZV2* level); *JV5* length; *St* IV length; spermatheca calyx length, and spermatheca calyx width (Fig. 1, Table 1).

### XGBoost training and computing

We used XGBoost to develop a classification system for target mite species and related species based on their morphological features. Among machine learning methods, XGBoost is the most efficient for implementing the gradient boosting decision tree algorithm from multiple decision trees, which are created successively. For each iteration, a tree enhances its predictive power by minimising the unexplained part of the last tree. First, we determined the number of decision trees through cross-validation. The original sample was randomly partitioned into five equally sized subsamples (Table S1). A single subsample and the other subsamples were retained for use as the validation and training data, respectively. Cross-validation was then performed five times, with each subsample used exactly once as the validation data. The number of decision trees allows the same level of performance to be achieved in training and validation. The number of decision trees was then used for the full dataset to create a final model, and key morphological features were selected for their relative importance. Next, we used ICE plots to indicate the determinative roles of these key features in classification. Plots in which one line represents one specimen indicate changes in predictions (of target species) that occur as a morphological feature change. We generated XGBoost and ICE plots by respectively using the R package “xgboost”^40^ and “pdp”^41^.

### Drawings

Hand-drawn illustrations (Fig. 1) were made under an optic microscope (Olympus BX51). These drawings were first scanned, then processed and digitized with Photoshop CS6 (Adobe Systems Incorporated, USA).

## Supplementary information

Supplementary file1
