# Supplementary material for: Semi-automated identification of biological control agent using artificial intelligence
Source: Sci Rep. 2020 Sep 3;10:14632. doi: 10.1038/s41598-020-71798-x (PMC7471324; doi:10.1038/s41598-020-71798-x)
Supplement: Supplementary file 1 — Supplementary file1 [file 41598_2020_71798_MOESM1_ESM.pdf]

Semi-automated identification of biological control agent using artificial intelligence

Jhih-Rong Liao[1], Hsiao-Chin Lee[1], Ming-Chih Chiu[2*], and Chiun-Cheng Ko[1*]

[1] Department of Entomology, National Taiwan University, Taipei City 10617, Taiwan

[2] Institute of Hydrobiology, Chinese Academy of Sciences, Wuhan City 430000, China

[*] mingchih.chiu@gmail.com; kocc2501@ntu.edu.tw

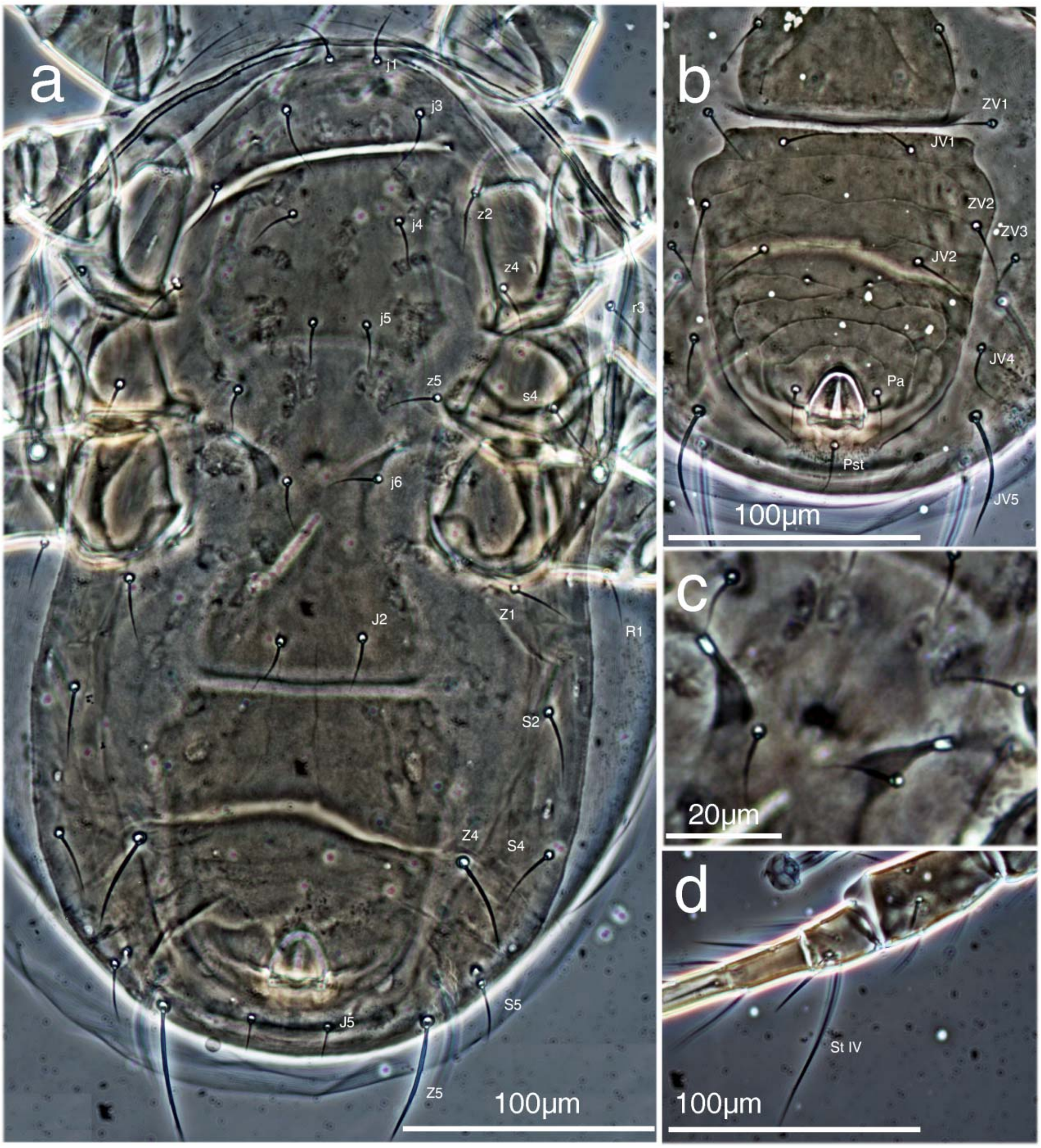

Figure S1. Measured variables of female phytoseiid mites in photomicrograph (a) dorsal shield, (b) ventral view, (c) spermatheca, (d) leg IV

Supplementary Table S1. Measurements of phytoseiid specimens (The column 'cv_idx' indicates the different subsamples for the cross-validation)

Scientific name	specimen	cv_idx	DSL	DSW.j6	j1	j3	j4	j6	J5	z2
Neoseiulus barkeri	88-0170	f4	351.63	192.07	17.37	23.07	16.02	17.43	12.94	22.47
Neoseiulus barkeri	KinmenSorghum	f3	361.52	206.07	17.79	23.32	18.5	18.78	11.95	19.81
Neoseiulus barkeri	88-Am-256	f4	346.72	184.08	18.75	24.4	17.29	19.22	11.95	21.09
Neoseiulus barkeri	HAL099C157	f4	389.28	216.39	21	18.32	19.91	18.78	12.05	21.49
Neoseiulus barkeri	HAL099C158	f2	383.25	213.62	19.64	25.42	17.43	18.78	12.83	22.35
Neoseiulus barkeri	TAL080B018	f4	325.66	173.77	14.32	18	15.42	16.28	10.86	18.11
Neoseiulus barkeri	TAL080B020	f1	332.24	182.99	16.36	20.3	14.31	18.78	10.67	18.09
Neoseiulus barkeri	TAL091B001	f4	358.79	188.42	17.3	24.15	16.39	18.78	10.22	21.19
Neoseiulus barkeri	TAL091B227	f5	388.49	187.45	15.54	21.54	14.98	17.81	11.75	17.39
Neoseiulus barkeri	TAL091B512	f1	323.61	171.07	16.12	20.64	13.39	16.03	9.9	16.61
Neoseiulus barkeri	TAL091C133	f3	340.49	176.9	14.91	21.34	17.05	16.26	11.15	19.12
Neoseiulus barkeri	TAL091I010	f5	353.87	185.65	16.57	21.47	14.84	17.62	9.94	18.19
Neoseiulus barkeri	TAL091I011	f5	348.1	192.69	12.79	19.68	14.43	16.05	10.5	15.92
Neoseiulus barkeri	TAL091I032	f3	359.66	194.81	16.98	22.02	14.44	16.71	9.45	18.21
Neoseiulus barkeri	TAL091I033	f3	356.55	199.67	17.32	23.6	16.14	16.05	11.12	18.24
Neoseiulus barkeri	TAL091I035	f4	352.16	205.59	19.58	25.32	19.41	20.72	11.25	20.89
Neoseiulus barkeri	TAL091S072	f1	368.6	201.19	18.72	28.78	17.89	19.22	12.31	22.69
Neoseiulus barkeri	TAL091S133	f2	347.54	185.83	18.76	23.96	18.54	18.79	9.92	20.2
Neoseiulus barkeri	TAL092G442	f2	384.34	216.2	20.64	23.74	16.02	19.3	13.31	24.33
Neoseiulus barkeri	TAL092G443	f5	380.74	206.57	18.97	22.87	17.76	18	11.8	22.13
Neoseiulus barkeri	TAL093B437	f3	371.24	205.39	19.12	25.45	19.53	15.9	13.56	25.61
Neoseiulus barkeri	TAL093B550	f3	364.58	208.01	18.27	21.5	16.21	16.82	10.71	20.94
Neoseiulus barkeri	TAL093B708	f3	375.06	210.95	17.98	24.92	20.28	18.78	10.49	22.76
Neoseiulus barkeri	TAL093B766	f4	381.92	217.38	19.94	27.91	17.51	18.81	11.3	23.73
Neoseiulus barkeri	TAL093B767	f1	376.41	218.73	20.18	24.11	17.98	15.66	11.01	20.15
Neoseiulus barkeri	TAL093B768	f4	393.26	225.56	23.49	28.17	17.87	16.56	11.23	23.2
Neoseiulus barkeri	19-XI-1986	f4	352.76	187.64	19.06	24.53	17.08	18.78	11.81	22.08
Neoseiulus barkeri	24-X-1988	f3	354.58	198.13	17.32	20.55	16.26	18.78	10.34	18.87
Neoseiulus barkeri	12-X-1988leftdata same	f2	339.93	186.41	18	21.44	15.59	15.01	11.03	19.86
Neoseiulus barkeri	12-X-1988rightdata same	f3	339.93	186.41	17.29	21.83	15.71	15.67	11.3	18.79

Neoseiulus barkeri	4-VI-1991	f4	369.75	204.62	19.39	25.39	17.28	18.17	12.98	21.73
Neoseiulus barkeri	88-Am-0686	f2	350.19	192.58	16.95	21.93	13.28	14.86	9.76	17.52
Neoseiulus barkeri	1561-11	f1	359.56	198.82	17.87	21.74	15.84	18.43	10.5	18.49
Neoseiulus barkeri	HAL095F297	f4	372.66	211.93	17.7	22.19	16.66	16.99	12.8	19.19
Neoseiulus barkeri	HAL095F309	f4	380.56	205.82	18.98	29.92	16.41	18.1	11.7	22.58
Neoseiulus barkeri	HAL095G063	f5	374.7	201.93	19.7	26.76	18.52	18.78	12.7	20.64
Neoseiulus barkeri	HAL095G064 up	f5	367.32	196.35	17.87	23.84	17.99	18.78	12.87	21.19
Neoseiulus barkeri	HAL095G064 down	f4	378.44	199.17	18.49	27.39	17.57	18.78	12.76	18.82
Neoseiulus barkeri	HAL095G064 right	f4	373.16	197.99	19.25	22.75	19.58	18.78	12.99	21.19
Neoseiulus barkeri	HAL099B487	f2	336.81	182.46	13.75	18.06	12.95	15.05	10.2	16.17
Neoseiulus barkeri	HAL100B075	f2	380.23	208.84	19.66	29.36	17.44	23.79	12.48	24.8
Neoseiulus barkeri	HAL101B144 left	f2	343.61	177.83	18.72	21.66	16.5	17.7	12.82	17.47
Neoseiulus barkeri	HAL101B144	f1	350.96	197.35	19.01	19.7	15.8	16.72	13.61	16.66
Neoseiulus barkeri	QAR101H001	f4	332.4	179.32	16.99	19.77	16.37	16.57	11.15	17
Neoseiulus barkeri	QAR101H013	f5	387.83	213.23	19.7	25.63	19.9	20.27	12.74	23.06
Neoseiulus barkeri	QAR101H014	f4	387.65	209.16	20.29	29.01	19	20.57	12.12	24.61
Neoseiulus barkeri	QAR101H023	f5	367.1	190.9	19.3	26.31	18.43	19.31	13.06	22.44
Neoseiulus barkeri	QAR102H022	f2	352.7	193.96	17.95	22.35	18.62	18.08	12.45	18.51
Neoseiulus barkeri	QAR102H029 left1	f3	358.76	198.63	19.16	28.03	19.88	20.39	12.77	22.41
Neoseiulus barkeri	QAR102H029 left2	f2	376.1	210.81	21.39	27.46	19.53	20.97	13.09	24.89
Neoseiulus barkeri	QAR102H029 right1	f2	358.36	192.04	19.35	25.21	19.06	19.48	13.88	23.93
Neoseiulus barkeri	QAR102H029 right2	f5	391.99	206.16	19.22	27.04	19.56	19.77	12.15	23.43
Neoseiulus barkeri	QAR102H029 right3	f2	352.72	196.31	19.38	24.05	18.67	19.93	13.82	22.91
Neoseiulus barkeri	QAR102H029 right4	f1	319.74	173.68	18.62	24.31	19.26	20.17	12.3	23.31
Neoseiulus barkeri	QAR102H036	f2	381.44	214.78	21.32	26.72	19.23	19.65	12.85	22.8
Neoseiulus barkeri	QAR102H038	f3	353.12	182.68	18.42	24.25	17.42	20.75	11.98	22.25
Neoseiulus barkeri	QAR102H050	f3	377.87	206.45	18.83	26.46	16.72	19.39	10.92	23.5
Neoseiulus barkeri	TARI-1	f1	407.21	225.74	19.29	25.23	17.43	18.77	11.95	21.19
Neoseiulus barkeri	TARI-2	f4	387.45	221	20.95	22.94	17.43	20.82	11.38	21.14
Neoseiulus barkeri	1213-5	f1	362.21	195.82	15.66	23.33	13.11	15.77	10.47	16.94
Neoseiulus barkeri	1285-3	f5	379.53	202.43	17.6	22.82	16.65	17.82	12.25	22.56
Neoseiulus barkeri	1561-3	f5	386.78	227.98	20.05	26.3	19.35	20.02	10.94	24.39

Neoseiulus barkeri	1561-4	f2	377.2	207.55	18.3	21.18	18.5	17.37	10.22	21.04
Neoseiulus barkeri	1561-5	f3	383.43	201.77	18.75	25.38	19.21	18.78	12.7	23.26
Neoseiulus barkeri	1561-6	f5	291.4	163.37	16.61	19.56	15.31	18.78	9.01	21.19
Neoseiulus barkeri	1561-7	f5	373.53	208.43	21.95	23.64	17.43	18.78	11.43	21.19
Neoseiulus barkeri	1561-8	f2	410.53	235.49	22.82	27.73	20.96	23.1	14.55	25.23
Neoseiulus barkeri	1561-9	f4	394.7	222.43	21.96	26.2	19.2	18.78	11.95	21.19
Neoseiulus barkeri	1561-10	f1	373.12	211.73	19.77	25.59	16.44	18.78	13.09	21.32
Neoseiulus barkeri	1561-12	f1	346.32	190.27	18.65	21.78	13.01	18.78	11.98	21.19
Neoseiulus barkeri	1561-13	f4	366.35	199.69	24.28	23.66	17.93	18.78	12.1	21.57
Neoseiulus barkeri	1561-14	f1	377.59	208.44	19.79	22.46	18.49	18.78	13.43	20.32
Neoseiulus barkeri	1561-15	f3	375.49	206.64	19.53	23.8	16.76	18.78	12.25	21.52
Neoseiulus barkeri	1561-16	f2	358.42	198.44	19.06	26.48	17.1	19.02	12.24	21.59
Neoseiulus barkeri	1561-17	f4	378.63	220.04	19.2	23.45	16.53	18.78	12.45	25.61
Neoseiulus barkeri	903-2	f1	379.05	216.77	20.23	26.01	17.14	18.74	12.65	21.53
Neoseiulus barkeri	Tainan monomacroseta	f1	361.35	197.38	18.75	24.99	17.84	16.61	12.76	21.88
Neoseiulus barkeri	GEI1 up	f1	343.87	192.48	15.16	25.63	16.24	22.29	11.94	20.74
Neoseiulus barkeri	GEI1 down	f1	352.42	185.43	18.18	25.78	18.25	21.26	13.8	20.91
Neoseiulus barkeri	GEI2 up	f4	360.55	190.91	17.21	27.6	17.98	24.29	13.24	22.57
Neoseiulus barkeri	GEI2 down	f3	365.95	191.06	21.87	26.37	19.22	18.51	12.69	23.72
Neoseiulus barkeri	GEI3	f5	359.54	200.98	21.87	24.55	18.24	18.44	12.68	20.86
Neoseiulus barkeri	GEI4	f2	363.84	199.07	18.97	24.8	18.08	16.77	10.99	22.95
Neoseiulus barkeri	GEI5 lowerright	f3	363.84	199.07	17.72	26.26	20.59	23.34	13.22	24.28
Neoseiulus barkeri	GEI5 below	f1	363.84	199.07	18.72	24.4	16.57	20.99	8.97	21.33
Neoseiulus barkeri	GEI6	f4	335.97	183.4	17.42	23.59	16.87	18.27	11.9	19.12
Neoseiulus barkeri	GEI7	f4	351.02	188.86	15.99	24.51	21.31	24.03	13.99	22.51
Neoseiulus barkeri	GEI8	f3	353.29	190.9	17.03	27.71	18.16	20.45	11.97	19.9
Neoseiulus barkeri	GEI10	f4	362.6	180.86	20.56	23.91	18.91	18.78	13.07	20.23
Neoseiulus barkeri	GEI11 left	f3	369.51	196.8	20.62	24.93	19.57	22.18	13.6	23.14
Neoseiulus barkeri	GEI11 right	f5	354.29	186.09	21.53	25.13	18.83	23.02	13.94	24.21
Neoseiulus barkeri	LFC2	f5	354.14	199.13	NA	17.27	15.29	20.36	10.45	17.51
Neoseiulus barkeri	LFC3	f5	366.3	203.48	17.31	22.55	18.02	18.32	8.76	20.76
Neoseiulus barkeri	LFC4	f1	369.24	200.65	12.49	15.82	15.27	10.65	7.41	10.39

Neoseiulus barkeri	LFC5	f3	356.16	185.86	15.55	21.13	12.82	15.97	4.94	16.5
Neoseiulus barkeri	GF1	f3	371.84	203.13	16.26	22.8	14.62	13.22	11.57	18.92
Neoseiulus barkeri	GF2	f3	380.21	226.09	18.19	19.1	16.9	16.01	11.68	17.22
Neoseiulus barkeri	GF3	f2	NA	192.93	NA	NA	NA	NA	11.19	NA
Neoseiulus barkeri	GF4	f2	356.54	204.04	16.92	19.76	14.93	15.11	11.28	19.46
Neoseiulus barkeri	GF5	f2	391.97	213.86	17.53	21.73	17.41	15.6	8.06	19.69
Neoseiulus barkeri	GF6	f2	364.3	211.26	17.99	21.31	15.65	NA	11.52	NA
Neoseiulus barkeri	GF7	f5	379.82	210.59	17.11	22.92	14.17	15.59	10.08	21.72
Neoseiulus barkeri	GF8	f3	348.49	204.92	15.37	21.67	15.87	NA	8.24	NA
Neoseiulus barkeri	GF9	f3	365.85	214.45	18.63	NA	16.92	14.15	9.05	21.25
Neoseiulus barkeri	GF10	f3	346.25	197.88	18.35	24.5	15.48	17.3	9.95	17.36
Neoseiulus barkeri	GF11	f3	366.37	195.11	15.19	19.03	17.16	15.58	10.84	20.95
Neoseiulus barkeri	GF12	f3	374.61	207.15	17.85	22.23	16.22	10.84	11.03	21.25
Neoseiulus barkeri	GF13	f4	367.34	197.31	12.04	18.83	16.01	13.87	10.5	21.34
Neoseiulus barkeri	GF14	f1	354.61	190.58	20.66	18.58	13.41	14.77	11.89	23.25
Neoseiulus barkeri	GF15	f5	356.26	190.53	15.21	18.26	16.22	NA	8.44	15.9
Neoseiulus barkeri	GF16	f3	370.57	208.12	19.23	19.89	14.87	NA	8.51	NA
Neoseiulus barkeri	GF17	f2	382.3	203.57	17.01	22.6	17.23	18.63	10.04	15.14
Neoseiulus barkeri	GF18	f5	364.9	210.08	18.08	19.19	15.9	15.29	12.28	NA
Neoseiulus barkeri	GF19	f2	381.83	207.34	16.77	21.4	16.89	NA	9.22	16.44
Neoseiulus barkeri	GF20	f3	348.32	205.33	17.33	15.86	14.32	NA	9.23	22.89
Neoseiulus barkeri	GF21	f2	363.8	198.97	14.45	15.27	14.32	NA	13.22	15.21
Neoseiulus barkeri	GF22	f2	369.02	202.34	14.62	23.78	14.92	16.6	9.08	16.64
Neoseiulus barkeri	GF23	f5	334.72	205.87	15.15	21.6	17.36	14.43	11.61	22.94
Neoseiulus barkeri	GF24	f3	353.68	191.58	14.83	16.12	14.86	13.92	13.78	19.69
Neoseiulus barkeri	GF25	f1	367.39	206.28	13.55	23.99	13.29	14.42	8.54	22.22
Neoseiulus barkeri	GF26	f3	358.04	204.15	22.49	22.26	19.18	16.19	9.35	18.08
Neoseiulus barkeri	GF27	f5	368.91	213.49	18.04	21.23	21.23	16.84	10.32	20.24
Neoseiulus barkeri	GF28	f5	372.25	203.46	16.64	17.28	15.17	NA	8.12	NA
Neoseiulus barkeri	GF29	f5	362.1	207.81	16.5	18.69	17.45	15.67	9.83	18.89
Neoseiulus barkeri	GF30	f5	361.15	190.45	17.32	16.69	NA	15.27	8.71	14.39
Neoseiulus barkeri	GF31	f5	383.85	210.09	14.7	22.91	16.75	18.08	11.86	20.31

Neoseiulus barkeri	GF32	f4	358.87	228.98	18.38	27.63	22	17.23	9.79	19.28
Neoseiulus barkeri	GF33	f5	365.04	200.59	14.38	19.9	15.81	15.29	9.49	22.26
Neoseiulus barkeri	GF34	f3	382.41	209.47	15.98	21.48	17.92	16.01	11.28	23.59
Neoseiulus barkeri	GF35	f3	359.54	199.63	18.17	23.61	19.98	16.2	12.32	20.02
Neoseiulus barkeri	GF36	f5	356.19	186.23	21.55	24.84	19.16	13.89	12.84	16.83
Neoseiulus barkeri	GF37	f2	380.32	195.11	14.01	22.83	NA	13.98	9.85	20.67
Neoseiulus barkeri	GF38	f3	370.42	208.97	17.2	19.85	15.91	NA	8.08	21.53
Neoseiulus barkeri	GF39	f1	369.17	197.05	15.44	21.12	15.55	14.68	11.89	20.1
Neoseiulus barkeri	GF40	f1	359.83	194.4	17.07	23.8	16.04	16.85	10.75	20.85
Neoseiulus barkeri	GF41	f4	377.38	187.21	NA	25.17	15.64	17.49	8.38	25.47
Neoseiulus barkeri	GF42	f4	352.65	189.68	NA	24.72	16.04	18.23	10.97	19.02
Neoseiulus barkeri	GF43	f5	370.98	203.84	14.26	19.26	12.93	16.13	9.5	16.71
Neoseiulus barkeri	GF44	f2	375.18	213.31	15.49	22.51	16.33	11.39	10.75	20.74
Neoseiulus barkeri	GF45	f3	370.92	195.18	16.83	23.99	16.36	17.85	12.86	24.06
Neoseiulus barkeri	GF46	f5	357.15	212.02	17.17	23.21	16.04	17.33	9.59	20.87
Neoseiulus barkeri	GF47	f2	375.02	208.6	18.55	22.2	19.45	15.91	10.3	18.46
Neoseiulus barkeri	GF48	f2	348.82	189.15	NA	20.08	17.27	17.91	9.59	19.51
Neoseiulus barkeri	GF49	f2	371.61	213.54	19.75	21.49	19.62	NA	11.15	22.74
Neoseiulus barkeri	GF50	f2	361.14	191.84	14.87	25.84	20.24	14.11	13.26	23.83
Neoseiulus barkeri	GF51	f2	390.43	195.89	17.17	20.15	17.29	19.94	12.3	22.81
Neoseiulus barkeri	GF52	f5	366.2	205.94	15.51	21.13	18.2	16.69	12.73	20.05
Neoseiulus barkeri	GF53	f1	363.81	198.46	15.63	NA	17.14	NA	11.42	16.5
Neoseiulus barkeri	GF54	f3	348.95	199.29	NA	18.84	20.49	16.37	12.54	18.66
Neoseiulus barkeri	GF55	f4	375.24	201.02	NA	NA	NA	NA	NA	14.87
Neoseiulus barkeri	GF56	f1	362.34	197.34	21.24	NA	17.57	15.44	10.14	21.47
Neoseiulus barkeri	GF57	f4	366.31	199.71	14.22	21.76	15.89	17.87	11.83	18.74
Neoseiulus barkeri	GF58	f4	352.93	203.46	16.77	25.4	14.75	12.56	14.27	22.69
Neoseiulus barkeri	GF59	f2	376.77	216.54	16.15	27.31	17.45	16.04	10.02	20.52
Neoseiulus barkeri	GF60	f1	382.02	194.61	20.12	NA	16.57	NA	10.14	18.74
Neoseiulus barkeri	GF61	f2	386.34	215.04	NA	22.58	15.44	11.96	9	20.7
Neoseiulus barkeri	GF62	f4	378.98	208.53	16.96	22.89	15.44	NA	10.42	20.94
Neoseiulus barkeri	GF63	f3	347.98	189.31	15.19	28.42	16.04	14.45	12.28	21.8

Neoseiulus barkeri	GF64	f2	387.84	196.17	21	23.83	18.57	14.2	8.4	20.89
Neoseiulus barkeri	GF65	f1	372.23	212	17.98	25.68	19.07	15.48	8.93	NA
Neoseiulus barkeri	GF66	f1	368.93	190.9	NA	20.83	14.23	15.35	10.98	21.16
Neoseiulus barkeri	GF67	f3	385.07	202.67	16.87	19.92	14.45	15.08	9.71	20.36
Neoseiulus barkeri	GF68	f2	336.33	206.76	16.15	21.78	20.03	19.13	10.74	18.81
Neoseiulus barkeri	GF69	f3	386.29	206.93	18.12	24.28	15.35	16.8	10.43	19.52
Neoseiulus barkeri	GF70	f2	369.49	205.4	14.23	15.96	13.48	NA	9	NA
Neoseiulus barkeri	GF71	f5	347.91	204.36	15.54	17.21	15.05	NA	8.48	14.57
Neoseiulus barkeri	GF72	f3	365.92	205.57	20.43	21.02	14.86	NA	10.75	NA
Neoseiulus barkeri	GF73	f2	357.81	204.72	18.73	23.75	18.16	15.77	11.12	NA
Neoseiulus barkeri	GF74	f1	375.65	209.88	16.86	NA	15.32	NA	8.24	NA
Neoseiulus barkeri	GF75	f1	371.53	209.01	13.98	20.51	16.61	15.99	10.9	15.19
Neoseiulus barkeri	GF76	f3	368.08	196.63	13.99	22.67	14.54	NA	11.71	NA
Neoseiulus barkeri	GF77	f4	362.02	198.29	18.16	22.3	15.91	15.75	11.76	22.8
Neoseiulus barkeri	GF78	f1	347.81	181.43	18.1	23.16	14.63	18.3	11.2	16.56
Neoseiulus barkeri	GF79	f3	365.47	210	16.69	20.96	17.74	18.66	11.7	22.86
Neoseiulus barkeri	GF80	f1	379.44	205.52	15.94	23.29	18	16.49	12.43	21.88
Neoseiulus barkeri	GF81	f2	377.9	212.35	15.68	23.92	16.53	13.19	9.87	17.85
Neoseiulus barkeri	GF82	f5	380.29	222.65	15.51	25.73	20.78	16.83	13.04	18.08
Neoseiulus barkeri	GF83	f1	371.99	204.17	NA	NA	12.99	NA	10.46	22.78
Neoseiulus barkeri	GF84	f1	360.39	197.8	18.97	22.93	18.58	14.23	10.8	19.17
Neoseiulus barkeri	GF85	f1	372.96	210.32	16.06	18.65	16.3	16.33	9.14	19.45
Neoseiulus barkeri	GF86	f2	375.17	193.42	17.34	19.44	NA	13.22	11.2	15.88
Neoseiulus barkeri	GF87	f1	359.34	201.24	15.39	26.85	16.82	13.24	12.88	18.56
Neoseiulus barkeri	GF88	f2	367.14	201.3	18.16	NA	NA	NA	10.06	22.5
Neoseiulus barkeri	GF89	f4	355.97	204.76	17.37	22.86	16.38	18.86	12.22	17.33
Neoseiulus barkeri	GF90	f3	371.73	203.08	20.31	19.3	16.3	14.23	9.99	21.35
Neoseiulus barkeri	GF91	f4	380.74	215.73	19.04	22.72	16.54	14.58	13.54	19.94
Neoseiulus barkeri	GF92	f1	389.55	225.84	20.86	18.83	17.65	15.53	14.78	21.61
Neoseiulus barkeri	GF93	f4	366.32	191.15	16.75	20.98	16.86	16.56	8.49	20.57
Neoseiulus barkeri	GF94	f5	363.81	194.74	18.36	20.12	15.35	18.04	11.02	22.49
Neoseiulus barkeri	GF95	f2	364.98	209.06	15.82	21.74	17.95	16.41	9.63	22.37

Neoseiulus barkeri	GF96	f1	365.88	206.25	14.49	22.24	17.46	11.72	9.93	17.82
Neoseiulus barkeri	GF97	f3	384.46	209.81	19.53	26.06	21.11	10.98	NA	23.31
Neoseiulus barkeri	GF98	f1	378.05	208.9	18.04	23.76	17.65	15.39	7.57	18.84
Neoseiulus barkeri	GF99	f2	358.34	205.05	19.01	22.78	16.83	14.96	13.09	20.94
Neoseiulus barkeri	GF100	f5	374.19	215.72	17.61	18.25	18.17	19.48	15.53	20.32
Neoseiulus barkeri	GF101	f1	372.36	221.65	19.09	27.32	18.42	16.16	10.45	17.18
Neoseiulus barkeri	GF102	f4	401.48	214.1	17.18	23.27	20.02	20.41	10.6	20.5
Neoseiulus barkeri	GF103	f4	355.71	210.39	23.6	25.55	20.09	14.49	12.94	NA
Neoseiulus barkeri	GF104	f1	351.13	188.23	22.21	22.27	16.47	19.75	9.41	23.27
Neoseiulus barkeri	GF105	f1	365.47	205.37	17.82	22.86	15.73	15.83	12.51	21.68
Neoseiulus barkeri	GF106	f3	374.04	201.86	17.72	24.7	17.11	NA	12.29	NA
Neoseiulus barkeri	GF107	f3	368.54	210.77	NA	18.17	17.12	20.49	11.2	19.88
Neoseiulus barkeri	GF108	f2	379.38	198.46	21.41	NA	16.56	16.99	11.33	20.79
Neoseiulus barkeri	GF109	f3	406.57	215.65	20.11	24.01	19.43	NA	10.62	21.39
Neoseiulus barkeri	GF110	f5	358.58	199.65	17.74	22.5	18.46	NA	12.8	19.05
Neoseiulus barkeri	GF111	f5	376.97	190.48	18.78	18.35	17.17	17.58	13.43	20.49
Neoseiulus barkeri	GF112	f4	385.6	226.33	14.88	25.27	14.37	13.64	12.08	21.07
Neoseiulus barkeri	GF113	f5	379.8	205.75	NA	24.59	16.19	17.56	9.68	22.7
Neoseiulus barkeri	GF114	f4	353.59	205.1	16.09	20.84	20.47	16.53	12.6	24.88
Neoseiulus barkeri	GF115	f3	361.38	205.98	19.64	26.08	17.47	17.18	11.93	22.52
Neoseiulus barkeri	GF116	f4	373.87	210.95	16.5	24.14	19.08	14.01	12.14	20.66
Neoseiulus barkeri	GF117	f4	391.59	199.68	NA	24.78	19.96	17.72	8.95	21.91
Neoseiulus barkeri	GF118	f1	379.15	203.04	15.42	23.84	16.81	NA	10.61	19.72
Neoseiulus barkeri	GF119	f5	380.4	216.66	15.16	26.03	19.08	14.9	13.57	21.42
Neoseiulus barkeri	GF120	f2	394.77	210.77	18.83	28.58	20.36	13.86	10.48	22.78
Neoseiulus barkeri	LFC6	f5	373.12	212.19	14.81	16.74	15.57	21.16	11.87	19.33
Neoseiulus barkeri	LFC7	f1	369.66	202.38	17.21	22.82	18.11	16.1	10.98	24.75
Neoseiulus barkeri	LFC8	f1	385.21	182.04	18.27	23.97	20.62	17.99	10.53	23.11
Neoseiulus barkeri	LFC9	f4	381.97	209.7	19.24	24.33	16.78	14.68	13.21	21.72
Neoseiulus barkeri	LFC10	f2	364.04	203.04	16.42	27.98	18.18	18.35	13.77	22.52
Neoseiulus barkeri	LFC11	f1	360.2	196.3	16.78	19.13	17.11	16.26	12.64	19.3
Neoseiulus barkeri	LFC12	f1	356.97	181.6	15.76	25.19	15.54	17.52	14.44	19.68

Neoseiulus barkeri	LFC13	f3	361.17	206.02	16.14	23.64	18.77	16.93	12.52	22.8
Neoseiulus barkeri	LFC14	f4	372.45	197.71	18.34	25.45	19.94	17.07	13.92	23.22
Neoseiulus barkeri	LFC15	f1	395.19	NA	18.78	24.48	19.17	22.39	12.92	25.62
Neoseiulus barkeri	LFC16	f1	356.6	192.19	15.01	17.07	16.73	15.58	12.99	20.8
Neoseiulus barkeri	LFC17	f5	366.61	193.91	18.5	18.65	15.92	14.76	12.29	21.68
Neoseiulus barkeri	LFC18	f2	350.14	199.53	21.54	24.15	20.98	13.99	10.5	22.4
Neoseiulus barkeri	LFC19	f3	384.43	196.78	17.53	22.58	18.14	16.77	16.01	25.37
Neoseiulus barkeri	LFC20	f5	365.6	202.52	18.92	24.34	16.99	16.63	12.68	18.22
Neoseiulus barkeri	LFC21	f5	347.49	198.34	17.95	19.65	17.98	18.05	9.9	20.48
Neoseiulus barkeri	LFC22	f4	349.22	186.83	18.58	23.28	16.43	12.25	7.81	23.82
Neoseiulus barkeri	LFC23	f2	342.87	199.91	15.83	20.9	19.3	18.59	14.94	24.57
Neoseiulus barkeri	LFC24	f1	362.46	206.89	17	22.67	19.13	14.92	9.88	15.08
Neoseiulus barkeri	LFC25	f3	355.55	198.87	NA	17.47	22.91	20.08	12.65	19.29
Neoseiulus barkeri	LFC26	f3	378.99	199.18	14.15	20.84	NA	15.72	14.11	22.8
Neoseiulus barkeri	LFC27	f1	344.27	186.4	19.73	20.37	14.23	16.63	13.14	17.57
Neoseiulus barkeri	LFC28	f2	366.1	182.11	17.34	22.86	16.8	17.69	11.86	21.9
Neoseiulus barkeri	LFC29	f5	353.54	192.91	14.85	18.14	12.89	13.65	9.56	20.27
Neoseiulus barkeri	LFC31	f3	381.38	206.72	17.86	23.38	18.86	12.69	10.22	20.45
Neoseiulus barkeri	LFC32	f3	347.08	181.36	16.89	18.94	17.54	21.23	11.29	22.51
Neoseiulus barkeri	LFC33	f2	356.81	200.47	15.87	23.64	20.36	15.2	12.37	20.53
Neoseiulus barkeri	LFC34	f4	371.32	201.53	20.56	18.53	17.78	16.6	10.66	19.07
Neoseiulus barkeri	LFC35	f5	353.39	202.16	15.83	23.76	16.78	16.72	10.04	22.39
Neoseiulus barkeri	LFC36	f5	382.75	201.44	20.98	27.66	20.18	17.45	16.73	27.24
Neoseiulus barkeri	LFC37	f5	375.48	201.62	20.4	23.08	20.5	16.75	14.87	20.36
Neoseiulus barkeri	LFC38	f3	376.2	197.6	17.7	20.67	18.53	17.25	11.8	20.42
Neoseiulus barkeri	LFC39	f5	366.85	201.05	12.27	23.41	16.75	19.75	11.54	NA
Neoseiulus barkeri	LFC41	f4	344.58	182.78	18.87	22.14	18.25	16.99	11.76	20.4
Neoseiulus barkeri	LFC42	f5	369.21	201.92	19.13	25.25	13.72	20.22	13.14	20.18
Scapulaseius anuwati	1014-5	f2	340	216	15.35	12.07	6.27	3.84	3.15	6.15
Scapulaseius anuwati	1056-7	f1	NA	223.6	9.63	12.56	4.54	5.66	7.84	8.52
Scapulaseius anuwati	1699-1	f2	348	234	12.01	13.69	5.01	8.97	8.29	10.34
Scapulaseius anuwati	15373	f1	337	229.33	14.06	12.65	6.39	7.39	8.31	10.73

Scapulaseius anuwati	16923	f4	341	227.42	11.65	12.56	6.75	7.65	8.26	8.69
Scapulaseius anuwati	21582	f3	330.75	238.19	11.87	12.29	4.94	7.37	8.64	11.66
Scapulaseius anuwati	21610	f4	329.44	215.43	11.69	12.63	5.44	6.6	8.11	9.62
Scapulaseius anuwati	anuwati-holotype	f2	317.01	218.33	10.09	8.99	5.65	6.49	6.44	8.22
Neoseiulus baraki	hal099b671	f4	424	184.96	16.08	16.99	13.23	13.62	7.87	13.48
Neoseiulus baraki	rice	f1	379	172.06	18.06	19.32	11.01	14.88	11.92	14.07
Neoseiulus baraki	tal092g562	f4	391	175	11.95	17.73	6.1	8.56	8.87	8.35
Neoseiulus baraki	tal092g563	f5	435	190	14.14	17.06	8.73	9	11.95	10.96
Scapulaseius cantonensis	1043-1	f2	373	256	26.14	15.63	8.29	10.66	6.11	9.24
Scapulaseius cantonensis	1503-1	f4	364	279	24.39	10.95	7.39	7.14	6.59	8.01
Scapulaseius cantonensis	1504-1	f5	378	262	29.65	10.32	8.01	5.63	6.33	10.3
Scapulaseius cantonensis	1615-1	f2	339	NA	27.8	13.26	8.27	6.56	5.1	10.01
Scapulaseius cantonensis	1615-2	f3	385	267.94	30.61	11.54	7.71	7.96	7.53	9.88
Scapulaseius cantonensis	1618-3	f3	373	NA	26.22	10.84	8.82	6.3	6.51	9.77
Scapulaseius cantonensis	1703-5	f2	375.12	265	23.7	9.71	5.83	7.08	7.34	8.24
Scapulaseius cantonensis	hal099b175	f1	362.67	238.55	25.4	11.94	7.22	7.92	5.25	6.84
Scapulaseius cantonensis	hal099b1777	f3	312.49	239.75	21.49	9.14	7.39	5.99	5.1	8.08
Scapulaseius cantonensis	HAL099b1779	f1	349.97	230.75	23.37	9.49	6.86	6.6	4.48	10.2
Amblyseius eharai	1513-5	f1	348	223.58	32.85	35.28	4.41	5.21	3.13	7.67
Amblyseius eharai	43844	f3	342	262	34.57	51.98	2.99	3.84	5.54	7.1
Amblyseius eharai	43996	f5	385	253	37.53	54.88	3.04	3.48	8.52	9.85
Amblyseius eharai	44026	f4	332	270	35.39	54.54	4.62	5.11	3.07	8.33
Amblyseius eharai	43846	f3	NA	NA	38.17	51.19	3.8	3.68	8.44	8.8
Amblyseius eharai	43882	f5	392.12	252.11	38.61	50.24	5.55	6.29	8.33	11.47
Amblyseius eharai	35521	f2	385.3	267.28	38.9	48.91	5.88	6.13	5.53	10.68
Amblyseius eharai	35796	f3	348.96	NA	33.53	47.44	6.05	5.34	6.83	10.38
Amblyseius eharai	15493	f3	381.8	255.19	36.86	48.51	3.06	7.89	3.55	15.65
Amblyseius eharai	1513-4	f5	386.87	234.35	37.98	49.77	2.71	5.83	6.7	9.14
Neoseiulus makuwa	makuwa-holotype	f3	333.39	199.97	15.85	17.43	7.23	6.25	4.52	11.29
Neoseiulus makuwa	makuwa0	f2	346.65	204.72	18.53	23.16	11.95	12.68	11.06	16.06
Neoseiulus makuwa	makuwa1	f4	328.92	199.75	17.84	19.52	11.53	12.51	10.06	17.62
Neoseiulus makuwa	makuwa2	f5	341.05	212.6	17.3	22.21	9.39	10.6	10.63	13.99

Neoseiulus makuwa	makuwa3	f2	324.87	196.2	16.69	23.84	11.43	13.48	11.1	16.56
Neoseiulus makuwa	an	f3	345	200.89	14.13	19.85	6.64	9.11	7.6	9.56
Neoseiulus makuwa	chuhisnag	f3	339.56	171.63	14.72	27.21	6.85	11.09	9.69	10.97
Neoseiulus makuwa	fenghung	f5	330	180.37	14.23	18.91	8.37	8.5	6.51	10.97
Neoseiulus makuwa	tal092n041	f5	306	170.27	14.13	15.99	6.15	8.16	6.77	6.35
Neoseiulus makuwa	tal092n047	f1	325	193.6	11.61	17.32	7.24	6.02	5.19	11.11
Scapulaseius okinawanus	okinawanus-holotype	f5	301	192	16.62	15.16	7.86	6.28	8.22	9.66
Scapulaseius okinawanus	88AM0615	f4	325	234	19.07	20.66	8.67	7.27	8.62	10.15
Scapulaseius okinawanus	1814-1	f5	334	212	19.74	NA	6.43	5.23	8.28	8.14
Scapulaseius okinawanus	1814-2	f4	326	201	20.65	NA	5.52	3.89	7.47	7.58
Scapulaseius okinawanus	44073	f5	323	203.96	NA	NA	9.12	9.2	6.92	14.1
Scapulaseius okinawanus	12086	f3	330	220.92	18.5	15.13	7.06	9.54	6.21	11.26
Scapulaseius okinawanus	12479	f4	331	212.27	16.97	15.19	8.83	8.16	6.73	10.89
Scapulaseius okinawanus	hal099b159	f5	386.69	196.17	22.83	14.17	5.22	5.4	5.87	9.81
Scapulaseius okinawanus	hal101b087	f5	319.13	226.31	19.18	18.3	5.21	7.11	6.72	8.65
Scapulaseius okinawanus	tal080b048	f4	375.88	235.75	20.33	15.66	10.14	11.08	9.15	12.5
Neoseiulus taiwanicus	taiwanicus-holotype	f1	351	137.89	6.88	12.73	7.37	8.01	6.78	10.75
Neoseiulus taiwanicus	rice1	f4	363	146.32	9.42	10.29	6.89	6.58	8.04	8.86
Neoseiulus taiwanicus	rice2	f1	354	153.53	10.81	10.68	7.05	9.34	7.89	8.28
Neoseiulus taiwanicus	rice3	f4	349	140.56	10.1	9.72	7.81	8.13	11.14	8.22
Neoseiulus taiwanicus	rice4	f2	331	146.57	9.33	12.87	8.05	6.56	8.05	6.6
Neoseiulus taiwanicus	rice5	f5	360	146.6	9.33	9.77	7.13	9.32	10.81	9.98
Neoseiulus longispinosus	1021-1	f1	326	183.66	18.45	NA	58.76	76.23	8.31	75
Neoseiulus longispinosus	1021-2	f2	347.21	196.81	17.04	54.4	65.72	83.96	10.36	98.35
Neoseiulus longispinosus	1021-3	f3	325.47	191.97	16.2	62.44	NA	84.83	8.05	62.8
Neoseiulus longispinosus	1218-2	f4	348	189	15.42	75.52	60.5	NA	NA	59.61
Neoseiulus longispinosus	1834-1	f5	339	191.29	17.75	62.79	52.01	73.69	11.39	73.23
Neoseiulus longispinosus	1834-6	f5	NA	178.45	22.22	68.04	NA	NA	17.43	70.03
Neoseiulus longispinosus	1834-7	f1	335	191.21	21.44	72.37	54.61	74.7	8.4	71.04
Neoseiulus longispinosus	34820	f4	335	185.02	19.9	54.12	52.61	73.93	9.85	52.9
Neoseiulus longispinosus	36526	f1	298	169.47	19.65	54.42	NA	58.26	8.4	55.03
Neoseiulus longispinosus	hal098b007	f5	340	187.24	19.28	74.54	NA	88.4	6.78	83.43

Scapulaseius tienhsainensis	1272-1	f4	340	212.96	15.5	22.55	8.9	8.63	6.65	10.75
Scapulaseius tienhsainensis	1985x17	f1	306	201.35	13.74	18.06	7.86	8.19	8.12	7.06
Scapulaseius tienhsainensis	26299	f2	330	207.12	16.4	21.96	10.14	12.51	7.26	9.7
Scapulaseius tienhsainensis	26330	f4	321	209.37	19.45	18.22	11.82	12.99	9.14	12.38
Scapulaseius tienhsainensis	26359	f1	330	213.68	13.78	21.03	13.31	13.37	10.11	NA
Scapulaseius tienhsainensis	26390	f4	324	215.73	22.76	24.7	12.7	11.35	9.65	11.6
Scapulaseius tienhsainensis	26420	f1	313	212.35	12.97	23.3	10.26	9.36	8.18	10.57
Scapulaseius tienhsainensis	lectuca	f1	331	219.28	19.86	21.61	9.04	10.56	7.83	10.37
Neoseiulus womersleyi	302-5	f2	342	191	19.91	56.61	54.58	65.7	11.53	56.31
Neoseiulus womersleyi	462-4	f3	335	173	18.8	41.85	49.72	70.69	7.77	62.74
Neoseiulus womersleyi	462-5	f4	330	180.68	22.85	47.97	41.78	55.56	8.59	52.85
Neoseiulus womersleyi	707-3	f4	345	181.75	17.96	58.1	44.66	58.56	8.4	54.7
Neoseiulus womersleyi	707-5	f1	338	184.98	16.27	53.23	50.23	59.42	9.03	57.77
Neoseiulus womersleyi	43860	f5	341	195	14.2	NA	NA	77.59	7.65	52.52
Neoseiulus womersleyi	44042	f4	342	175.26	17.75	54.18	44.41	59.36	8.54	63.76
Neoseiulus womersleyi	12055	f4	361.53	195.03	23.62	62.93	60.2	77.43	10.03	69.51
Neoseiulus womersleyi	tari kinmen	f2	325.35	184.93	NA	62.36	NA	NA	NA	65.33
Amblyseius alpinia	157-4	f1	342	199.62	19.18	44.73	4.87	5.72	7.9	15.24
Amblyseius alpinia	HAL098B464	f4	361	214	30.6	51.65	7.54	9.51	6.59	18.08
Amblyseius alpinia	HAL098B481	f4	367	208	23.77	46.91	6.96	8.32	8.25	11.43
Amblyseius alpinia	HAL099B849	f3	360	193	28.65	45.9	4.76	7.21	7.62	6.67
Amblyseius alpinia	HAL099B857	f1	NA	NA	29.42	51.85	NA	NA	7.03	12.14
Amblyseius alpinia	TAL074C005	f1	416.88	240.66	31.29	55.45	8.51	8.57	6.85	15.75
Amblyseius alpinia	TAL077G006	f5	388.73	230.18	29.89	49.89	5.81	7.62	6.3	12.6
Amblyseius alpinia	TAL079B024	f5	NA	NA	24.93	42.76	5.19	3.82	5.14	11.16
Amblyseius alpinia	TAL087K089	f1	409.33	NA	32.74	54.08	8.62	5.87	6.28	16.02
Amblyseius bellatulus	TARIZingiber	f5	378.86	222.02	26.6	36.44	6.77	4.1	3.98	10.77
Amblyseius bellatulus	TARIRorippa	f1	361.07	196.82	25.65	37.89	7.36	7.53	8.07	9.5
Amblyseius bellatulus	TayuanAlliumsoil	f4	449.28	250.15	30.6	46.28	6.47	6.95	6.51	10.21
Amblyseius bellatulus	Polygonum	f2	386.66	207.58	25.91	36.22	7.03	6.94	6.55	10.26
Amblyseius bellatulus	TARImelongena	f2	354.86	202.8	29.57	39.23	6.12	7.03	4.68	8.4
Amblyseius bellatulus	TARIoryza	f1	395.22	215.2	32.39	45.32	12.97	7.25	7.97	10.39

Amblyseius bellatulus	Ampelopsis	f1	352.5	191.77	25.56	38.2	7.33	8.15	8.8	9.3
Amblyseius bellatulus	Psidium	f1	385.02	216.72	27.09	40.25	4.91	7.63	7.59	10.68
Amblyseius bellatulus	Lagenaria	f1	397.32	211.74	28.61	41.65	4.81	5.94	4.98	9.01
Amblyseius bellatulus	Neotype	f4	382.23	202.28	23.3	33.57	4.59	6.19	5.03	10.5
Amblyseius herbicolus	1304-2	f1	346	230.29	27.02	50.86	5.67	3.97	5.92	4.4
Amblyseius herbicolus	1391-1	f5	359	247	36.53	38.92	7.78	11.06	7.57	8.1
Amblyseius herbicolus	1513-3	f5	364	243	34.72	38.71	4.72	3.51	4.22	7.31
Amblyseius herbicolus	1528-1	f5	352	232	34.18	30.62	3.09	4.56	4.56	8.27
Amblyseius herbicolus	1528-2	f2	319	225.53	30.41	30.96	4.35	4.74	4.74	7.35
Amblyseius herbicolus	1528-5	f1	322	213.21	34.05	41.86	3.44	5.51	5.45	5.62
Amblyseius herbicolus	43897	f3	352	244.86	35.82	40.18	4.78	4.44	7.2	8.97
Amblyseius herbicolus	43931	f5	338.35	233.87	25.9	36.5	3.72	5.31	5.65	8.45
Amblyseius herbicolus	21186	f2	NA	NA	31.99	38.59	5.26	3.08	4.87	9.18
Amblyseius herbicolus	23774	f2	369.39	262.91	33.55	38.79	3.44	3.51	6.47	6.83
Amblyseius tamatavensis	12540	f4	366	236	32.04	50.88	6.94	5.16	8.15	5.99
Amblyseius tamatavensis	88-2615	f5	355	246	28.88	49.25	3.65	8.34	7.34	5.68
Amblyseius tamatavensis	12086	f2	383	242	32.76	51.9	5.37	4.13	5.75	4.17
Amblyseius tamatavensis	19921001tari	f3	348	235	28.29	49.08	4.46	3.43	5.16	4.47
Amblyseius tamatavensis	hal097b685	f5	351	214	30.95	47.62	5.42	4.87	4.13	4.87
Amblyseius tamatavensis	hal097b688	f2	380	220.91	32.07	50.3	4.12	4.61	4.09	3.86
Amblyseius tamatavensis	hal097b760	f4	NA	NA	28.55	49.4	4.6	5.42	5.9	6.07
Amblyseius tamatavensis	hal097b802	f4	NA	205.82	34.65	51.3	6.18	5.14	6.94	7.36
Amblyseius tamatavensis	hal097b682	f3	371.09	230.87	37.32	51.44	4.34	5.19	7.34	5.19
Amblyseius tamatavensis	tari1085xii27	f1	368.98	237.34	29.96	47.55	4.12	6.79	4.17	6.17
Euseius aizawai	1019-4	f3	335.53	243.76	26.92	25.89	5.53	9.66	4.86	15.09
Euseius aizawai	1019-5	f2	359.35	233.58	26.99	29.08	8.57	10.23	4.78	20.75
Euseius aizawai	1262-4	f2	359.14	265.41	32.76	29.11	9.4	11.5	6.04	18.48
Euseius aizawai	1283-1	f2	343.62	237.34	NA	24.29	9.22	10.02	5.91	16.69
Euseius aizawai	1292-1	f5	351.98	249.61	29.84	32.55	10.37	7.88	5.88	22.25
Euseius aizawai	1292-2	f2	336.19	248.23	34.16	39.8	9.47	12.14	6.34	19.09
Euseius aizawai	1293-1	f2	358.09	242.52	NA	26.81	7.71	7	NA	21.6
Euseius aizawai	1461-1	f4	287.58	208.5	NA	NA	9.24	8.34	5.63	11.94

Euseius aizawai	1513-1	f3	301.89	205.79	26.9	24.76	7.86	10.64	6.87	16.13
Euseius aizawai	1513-2	f5	307.71	228.8	NA	33.18	9.35	10.53	6.13	17.57
Euseius circellatus	747-5	f4	251	150	19.42	19.19	15.96	14.07	4.15	19
Euseius circellatus	tonpu-2	f5	273.53	162.68	20.01	19.62	17.41	14.67	4.75	21.49
Euseius circellatus	1259-9	f5	264.11	163.7	24.05	19.69	15.88	16.17	5.62	20.74
Euseius circellatus	tonpu-1	f5	275.44	159.74	19.92	21.55	16.57	14.33	5.22	21.77
Euseius circellatus	1670-2	f2	268.55	154.44	19.89	20.35	15.27	14.53	3.82	25.51
Euseius circellatus	1677-2	f4	278.35	170.01	20.61	24.4	17.08	17.87	5.28	23.59
Euseius daluensis	700-1	f3	293.07	208.23	23.45	19.49	5.58	5.88	2.72	8.75
Euseius daluensis	700-3	f2	293.02	203.76	26.72	15.59	3.64	4.97	2.42	7.43
Euseius daluensis	700-4	f3	285.22	203.85	24.86	14.72	4.05	5.57	2.37	7.96
Euseius daluensis	1616-3	f1	333.4	225.81	27.58	24.62	7.31	6.72	4.03	11.24
Euseius daluensis	tal074C10-1	f5	332.93	233.82	29.77	26.3	8.08	6.93	4.9	12.63
Euseius daluensis	tal074C10-2	f3	334.68	227.32	31.97	26.81	6.53	8.52	3.31	13.35
Euseius daluensis	daluensisholotype	f3	293.07	208.23	23.45	19.49	5.58	5.88	2.72	8.75
Euseius macaranga	390-8	f4	259.22	143.13	20.87	24.74	16.98	13.07	2.91	23.77
Euseius macaranga	609-3	f1	240.92	139.4	18.35	18	15.27	12.6	4.83	17.56
Euseius macaranga	609-4	f5	252.64	152.66	18.11	17.96	13.76	8.1	2.59	17.67
Euseius macaranga	737-3	f1	249.29	137.28	20.03	21	13.49	13.9	5.6	22.02
Euseius macaranga	946-5	f2	252.85	160.23	20.82	18.65	13.21	11.36	4.61	NA
Euseius macaranga	962-4	f1	278.11	151.04	21.51	17.87	16.17	14.91	3.24	22.41
Euseius macaranga	1111-5	f2	278.9	159.49	22.84	21.95	17.43	15.34	3.2	20.21
Euseius macaranga	1135-8	f3	275.33	160.2	22.78	25.63	15.66	15.13	4.49	27.88
Euseius macaranga	1190-7	f3	268.05	157.41	17.6	22.53	17.06	14.14	2.81	22.95
Euseius macaranga	1479-1	f3	255.34	149.83	17.61	19.01	14.22	12.32	4.35	18.89
Euseius macaranga	390-2type	f4	240.37	146.93	20.17	27.21	17.44	13.59	4.96	23.86
Euseius ovalis	1105-2	f3	358.07	242.91	35.9	8.14	3.79	4.98	3.26	9.17
Euseius ovalis	1109-1	f1	385.13	239.21	36.79	8.91	4.76	6.58	3.36	7.11
Euseius ovalis	1186-3	f4	363.05	256.47	31.22	10.94	6.06	4.82	2.55	3.98
Euseius ovalis	1190-11	f2	366.5	231.1	32.68	7.97	5.34	6.55	5.25	4.56
Euseius ovalis	1240-1	f2	365.18	244.39	32.47	8.17	4.33	5.42	5	8.62
Euseius ovalis	1244-1	f5	374.58	248.95	32.6	12.02	4.1	4.73	4.98	7.06

Euseius ovalis	1245-3	f2	390.53	260.1	31.32	11.48	5.04	8.4	6.06	11.34
Euseius ovalis	1315-2	f3	369.43	NA	32.96	10.03	2.4	2.87	5.1	8.46
Euseius ovalis	1201-6	f3	400.77	280.6	33.93	9.24	3.4	6.95	3.85	6.18
Euseius ovalis	1249-1	f1	388.96	255.9	36.09	11.44	5.31	7.22	2.87	7.96
Euseius paraovalis	1324-3	f3	321.12	217.33	28.7	5.34	5.38	5.83	3.77	5.8
Euseius paraovalis	1207-1	f1	339.62	243.17	35.17	6.41	4.61	5.49	4.01	5.75
Euseius paraovalis	462-14	f4	341.8	252.28	31.75	5.44	5	6.56	4.81	5.9
Euseius paraovalis	445-3	f4	336.08	237.19	34.7	4.16	3.85	5.04	3.87	5.31
Euseius paraovalis	425-1	f5	343.71	249.71	33.28	4.08	4.53	5.85	4.56	4.82
Euseius paraovalis	445-2	f3	346.61	246.08	32.88	5.51	3.71	5.31	4.58	3.52
Euseius nicholsi	388-1	f3	350.21	252	29.46	28.48	10.85	4.8	3.91	14.44
Euseius nicholsi	942-6	f2	349.84	242.84	33.5	25.68	6.07	9.65	5.76	15.46
Euseius nicholsi	holotype	f1	360.23	256.17	34.49	27.72	10.25	NA	6.38	16.59
Euseius oolong	1688-2	f3	360.77	266.42	26.93	23.26	7.55	6.86	2.68	13.8
Euseius oolong	1688-3	f3	346.48	254.44	27.12	20.75	6.75	6.14	4.92	13.43
Euseius oolong	43889	f4	332.52	243.93	31.37	25.16	10.1	10.27	4.23	15.38
Euseius oolong	43859	f5	323.85	239.04	24.03	20.03	10.38	11.31	4.1	17.65
Euseius oolong	43890	f1	356.86	236.07	NA	24.85	8.21	10.29	5.49	17.17
Euseius oolong	1688-1holotype	f4	327.68	236.6	31.97	19.61	7.47	9.46	5.84	12.33
Euseius sojaensis	1585-4	f5	334.42	227.58	33.63	27.99	8.14	6.46	4.99	20.99
Euseius sojaensis	1585-5	f1	338.3	239.69	31.14	19.69	8.68	10.92	4.4	16.24
Euseius sojaensis	1591-7	f5	366.42	211.28	NA	NA	13.73	9.9	6.6	18.51
Euseius sojaensis	1585-1	f2	332.43	239.82	NA	20.88	7.26	7.76	4.59	18.1
Euseius sojaensis	1113-1	f2	329.04	232.63	NA	NA	6.55	9.8	4.83	18.34
Euseius sojaensis	1283-2	f2	325.93	227.85	NA	25.31	6.28	8.72	5.49	15.49
Euseius sojaensis	1471-1	f2	314.27	229.43	24.34	23.73	7.34	10.3	6.83	15.77
Paraamblyseius formosanus	formosanustype	f1	350	263	10.71	19.99	13	23.15	6.3	19.62
Paraamblyseius formosanus	680-1	f2	321	245	8.71	19.36	20.15	31.42	11.31	19.58
Paraamblyseius formosanus	18323	f3	327	256	9.33	16.64	14.45	20.48	13.58	NA
Paraamblyseius formosanus	354-1	f3	292.61	222.09	8.31	13.89	12.62	NA	9.87	18.67
Proprioseiopsis asetus	24x1988	f3	350	229	18.3	23.81	4.81	6.02	6.17	5.11
Proprioseiopsis asetus	88-2450	f1	369	240.83	19.83	28.22	5.78	6.28	5.98	10.49

Proprioseiopsis asetus	88-am-0625	f3	356	227	18.39	28.84	6.5	5.86	7.38	9.42
Proprioseiopsis asetus	88-am-0659	f3	373	NA	15.89	31.26	5.61	5.97	6.7	11.5
Proprioseiopsis asetus	88-am-1274	f5	336.88	224.33	13.49	24.55	4.58	5.95	5.57	7.33
Proprioseiopsis asetus	1285-1	f4	375.89	240.5	20.02	35.37	5.72	6.19	8.49	14.34
Proprioseiopsis asetus	1561-18	f4	373.14	249.01	20.71	35.19	6.01	5.95	5.71	12.66
Proprioseiopsis asetus	1561-19	f5	423	279.5	22.7	35.54	5	4.2	7.27	12.3
Proprioseiopsis asetus	1561-20	f4	390.41	218.51	20.84	28.04	6.12	6.91	6.75	14.83
Proprioseiopsis asetus	tal091h097	f5	352.04	NA	18.52	34.72	7.44	5.88	6.89	13.79
Proprioseiopsis ovatus	587-6	f4	400	307	28.62	60.96	7.63	6.54	8.46	29.86
Proprioseiopsis ovatus	Meishan1	f1	308	247.15	24.85	47.58	5.35	4.94	6.59	28.61
Proprioseiopsis ovatus	Meishan2	f3	336	306.57	25.19	65.5	7.33	3.84	8.43	29.61
Amblyseius pascalis	Sichun	f5	382	229	32.65	47.83	10.66	6.85	6.84	5.96
Amblyseius pascalis	guizhou	f5	410	216	35.35	48.1	8.24	4.89	10.15	9.11
Phytoscutus salebrosus	823-11	f1	427	454.01	23.12	43.25	8.04	15.91	8.29	31.25
Phytoscutus salebrosus	896-3	f4	453	357.75	21.7	NA	7.37	12.86	9.16	31.59
Phytoscutus salebrosus	taoiholotype	f2	438	358	18.12	51.75	6.31	9.46	8.31	23.44
Phytoscutus salebrosus	taoiparatype1	f4	373	399	23.18	56.81	6.83	10.51	12.4	15.52
Phytoscutus salebrosus	taoiparatype2	f4	413	402	22.57	NA	12.19	15.28	14.15	18.64
Phytoscutus salebrosus	taoiparatype3	f4	419	399	19.81	49.68	7.9	8.21	11.09	14.84
Paraphytoseius chihpenensis	holotype	f5	286	158.08	40.09	97.14	4.45	5.12	4.99	7.45
Paraphytoseius chihpenensis	87-0519	f3	287	160.91	30.53	92.48	5.42	7.9	7.28	7.91
Paraphytoseius chihpenensis	87-0528	f3	266	161.68	37.13	92.19	4.97	5.59	6.59	7.03
Paraphytoseius chihpenensis	87-0529	f5	274	157.28	31.2	87.86	4.29	4.35	6.91	6.3
Paraphytoseius chihpenensis	87-0517	f3	308.09	164.14	36.68	96.96	4.83	5.76	5.43	7.7
Paraphytoseius chihpenensis	87-0526	f5	267.76	146.85	27.25	84.35	5.36	5.82	7.99	7.09
Paraphytoseius chihpenensis	87-0517	f5	278.82	159.62	36.75	79.22	4.48	8.09	5.94	6.77
Paraphytoseius cracentis	1791-10	f2	292.85	155.62	30.88	77.77	5.53	4.3	2.95	5.85
Paraphytoseius cracentis	1791-11	f1	274.42	145.62	30.86	75.49	2.76	3.02	2.24	5.85
Paraphytoseius cracentis	1791-12	f2	289	151.71	28.62	82.61	3.16	3.86	3.9	6.22
Paraphytoseius cracentis	1791-13	f1	286	144.18	27.84	80.02	2.76	4.18	3.58	5.48
Paraphytoseius cracentis	1791-14	f5	283	156.18	22.78	81.12	3.86	4.56	3.02	8.17
Paraphytoseius cracentis	1791-16	f1	278	128	32.22	84.9	1.95	5.18	3.16	7.92

Paraphytoseius cracentis	1792-3	f2	287.33	138.66	29.7	83.02	3.41	3.45	3.17	4.89
Paraphytoseius cracentis	1792-4	f2	283	137.3	33.44	84.76	2.03	3.02	2.9	7.75
Paraphytoseius cracentis	1806-1	f3	290.67	154.57	29.88	82.14	2.33	5.02	4.17	4.53
Paraphytoseius cracentis	1806-6	f4	292.31	159.57	33.98	90.43	5.99	4.3	4.06	5.34
Paraphytoseius orientalis	1785-6	f2	251.32	NA	129.71	23.15	78.77	4.46	2.85	9.02
Paraphytoseius orientalis	1787-7	f1	277	150.49	133.17	36.15	84.72	4.36	2.88	5.4
Paraphytoseius orientalis	1787-9	f4	270.68	145.75	139.31	39.98	85.03	4.66	3.34	5.84
Paraphytoseius orientalis	1787-10	f5	263.8	141.97	132.83	NA	79.99	4.55	3.62	6.46
Paraphytoseius orientalis	1853-3	f1	290.88	146.5	126.93	35.75	82.38	4.03	4.29	6.35
Paraphytoseius orientalis	1853-4	f5	287.1	151.61	131.6	35.16	79.25	2.72	5.68	4.8
Paraphytoseius orientalis	1853-6	f3	256.31	159.96	145.29	33.83	80.88	2.38	3.97	3.73
Paraphytoseius orientalis	1853-7	f4	285.15	146.53	140.75	38.45	82.52	2.33	2.01	4.67
Paraphytoseius orientalis	1853-10	f2	285.76	150.85	135.9	32.67	81.83	6.6	3.73	5.02
Paraphytoseius orientalis	1853-2	f4	300.16	146.32	138.48	36.03	86.71	2.82	3.4	4.03
Paraphytoseius hualienensis	hualienensistype	f5	280	126.76	36.41	96.34	4.39	6.86	5.37	4.49
Paraphytoseius hualienensis	paratype880534	f4	272	157.93	28.05	77.97	3.9	4.04	5.65	8.33
Paraphytoseius hualienensis	88pa0014	f1	263	147	26.96	78.23	4.09	6.2	6.43	7.09
Gynaeseius liturivorus	liturivorustype	f2	368	225.78	17.51	13.73	5.3	4.06	5.49	6.14
Gynaeseius liturivorus	11720	f1	386	251	18.64	16.96	8.91	13.53	6.47	13.89
Gynaeseius liturivorus	11749	f3	381	195	17.37	16.71	11.9	6.6	8.2	10.55
Gynaeseius liturivorus	11780	f2	348	215	21.93	20.9	11.79	10.64	4.93	8.48
Gynaeseius santosoi	1646-2	f4	405.5	246.6	29.6	14.67	7.81	6.58	5.82	9.21
Gynaeseius santosoi	43877	f4	395.47	236.1	28.45	15.04	7.31	6.09	7.43	11.2
Gynaeseius santosoi	santosoitype	f2	358.35	254.71	27.21	14.56	7.26	8.21	5.91	9.12
Okiseius subtropicus	1096-3	f4	283.96	NA	22.55	19.79	11.81	26.25	5.01	21.52
Okiseius subtropicus	1169-4	f3	319	185.03	22.56	13	12.55	26.72	5.89	25.36
Okiseius subtropicus	subtropicustype	f2	273.66	158.39	17.06	16.64	9	21.93	6.11	18.45
Okiseius subtropicus	HAL099B710	f1	297	173	18.38	20.38	13.72	25.49	5.58	21.72
Okiseius subtropicus	HAL099B711	f2	294	174.6	18.56	20.29	12.29	26.84	9.15	23.02
Okiseius subtropicus	HAL099B714	f2	319	181.41	19.63	22.55	14.08	24.63	9.05	20.89
Okiseius subtropicus	HAL101B205	f5	307	175.23	18.52	22.42	12.81	22.55	5.87	20.89
Okiseius subtropicus	HAL101B206	f3	328	196.19	19.19	20.58	14.88	26.44	8.13	22.04

Okiseius subtropicus	TAL078B018	f1	319.17	188.29	20.05	24.41	13.04	22.17	8.3	23.19
Okiseius subtropicus	TAL078B023	f4	318.5	168.68	22.67	23.48	15.41	21.32	4.46	25.18

z4	z5	Z1	Z4	Z5	s4	r3	R1	VSL	VSW.ZV2	JV5	TaIV	Calyx.L	Calyx.W
25.66	18.31	20.61	32.71	51.08	25.63	22.73	21.16	130.51	112.73	42.7	72.09	21.73	8.7
21.45	18.3	23.5	36.76	53.52	26.95	18.5	19.31	127.08	110.47	55.6	66.98	17.15	8.91
21.45	21.28	23.58	33.19	53.52	27.42	20.42	21.43	116.5	100.52	47.57	64.31	17.94	10.55
21.45	18.3	22.26	36.76	59.06	26.31	23.9	19.64	132.54	110.08	55.53	70.24	18.45	6.98
26.83	18.3	22.26	36.76	57.54	26.31	20.13	21.13	135.91	109.37	55.83	68.74	16.42	9.76
16.48	18.3	18.22	30.42	41.82	21.48	16.89	14.49	110.26	93.76	40.41	64.67	18.51	5.82
18.24	14.26	17.93	36.76	43.65	25.86	18.12	15.15	115.05	94.37	44.04	64.72	19.14	9.11
21.45	17.53	22.26	36.76	53.52	26.31	19.61	16.04	125.97	103.75	49.01	70.13	20.63	5.94
17.41	15.92	16.97	37.59	54.29	23.73	19.11	14.98	119.98	100.46	47.11	67.16	20.97	7.57
18.84	16.43	19.94	31.63	41.91	21.66	16.52	14.24	107.1	95.88	41.58	56.94	20.33	5.42
20.91	15.57	20.32	33.31	52.49	21.28	18.96	15.87	117.9	95.67	42.22	62.83	18.3	7.52
17.74	18.49	17.19	33.08	50.67	23.37	16.67	13.59	123.21	104.92	44.87	66.49	16.24	4.96
17.55	14.95	18.75	33.32	50.71	23.06	16.62	14.47	124.52	107.09	42.07	72.27	19.32	8.38
18.62	16.52	18.73	33.99	47.53	25.18	18.49	14.64	132.44	107.07	48.03	68.82	23.06	9.34
19.54	16.73	19.3	34.39	50.08	25.26	18.69	16.8	127.21	110.22	44.55	54.31	21.46	8.69
21.95	16.84	22.97	37.43	49.47	24.23	17.13	18.88	129.84	104.36	43.78	69.87	19.57	6.63
21.54	20.24	23.08	36.62	56.27	30	24.9	22.46	131.26	107.97	53.93	67.09	27.41	8.44
17.16	20.32	24.31	35.96	50.16	24.56	22.37	19.14	118.7	96.33	46.93	62.45	19.97	8.11
21.45	18.1	20.94	35.4	53.58	26.31	22.14	18.7	134.11	115.01	47.41	70.23	25.9	12.14
19.08	17.65	23.21	35.26	48.79	29.26	24.15	19.94	130.22	106.53	45.8	71.79	14.92	11.15
23.32	19.64	25.31	35.85	52.42	28.82	18.74	19.46	136.06	109.43	47.45	68.45	22.63	10.99
20.47	16.15	19.77	34.77	49.77	24.53	19.46	15.41	127.2	107.85	50.85	63.57	18.61	6.63
21.05	18.15	20.48	39.41	54	22.76	23.24	20.79	137.16	111.38	52.47	60.3	21.36	9.28
24.04	18.71	22.43	41.08	58.33	27.82	19.36	19.93	136.95	111.21	52.32	73.43	29	12.93
22.45	18.51	25.12	42.86	59.64	26.68	19.8	19.45	130.45	115.53	50.32	78.26	18.59	10.8
24.44	20.97	25.54	40.82	58.66	30.58	23.88	19.73	146.14	113.73	53.97	72.22	18.51	11.12
19.48	17.53	21.48	38.11	50.97	27.78	18.59	16.98	120.33	98.19	47.89	68.61	16.48	7.1
16.11	17.53	20.16	33.56	50.38	23.5	16.96	13.94	125.42	105.09	42.24	67.39	20.93	6.51
19.43	15.27	20.67	35.7	51.75	23.14	19.55	15.91	121.84	96.41	46.27	71.73	18.9	7.23
19.3	15.54	20.74	36.39	51.75	22.96	18.72	15.03	121.84	96.41	46.27	71.73	19.09	5.51

21.93	18.14	20.74	39.42	55	27.82	20.5	20.51	131.31	109.85	47.81	67.18	19.86	9.99
17.52	14.85	20.82	33.94	55.97	21.86	19.43	17.03	121.82	97.15	48.01	67.57	21.19	6.13
19.88	15.46	19.75	35.07	58.4	25.34	22.06	18.8	133.01	111.44	50.32	69.79	16.63	7.14
20.16	17.45	21.21	41.94	59.06	24.66	21.61	17.86	141.02	113.33	53.33	67.49	19.09	7.33
24.91	17.92	25.07	41.61	57.9	28.27	21.91	18.81	129.84	105.89	53.83	68.94	22.72	8.24
21.45	18.06	21.4	38.61	56.24	26.31	24.54	21.56	128.2	108.84	51.11	66.35	14.67	7.56
21.45	17.7	25.95	36.55	53.51	26.31	19.95	19.07	120	103.69	43.91	69.7	22.52	7.05
21.45	16.56	23.2	37.98	55.08	26.31	22.53	22.36	126.84	109.51	47.43	63.08	23.45	8.18
21.45	19.76	26.04	41.15	57.61	28.56	26.07	24.3	131.72	106.58	51.33	63.55	17.67	8.05
15.65	14.22	16.58	31.22	43.46	18.14	18.15	14.54	117.11	97.32	39.53	60.94	21.59	6.89
25.73	19.8	24.21	42.07	55.44	31.45	24.67	21.26	129.19	112.03	49.39	70.58	22.23	6.98
16.83	17.35	19.93	36.72	51.64	19.68	17.13	16.45	115.27	103.07	39.24	62.1	19.94	7.15
16.74	16.62	17.48	35.78	50.72	22.37	17.88	16.49	125.49	110.55	43.93	66.29	21.12	7.13
18.57	16.69	20.65	39.12	50.93	22.69	19.93	17.8	114.98	103.08	45.81	68.04	18.39	6.85
23.77	19.96	26.58	39.28	56.54	32.64	21.81	20.52	133.53	108.48	49.74	68.28	19.79	9.19
25.44	18.54	23.47	36.76	60.97	28.7	23.94	21.96	135.6	108.16	52.33	70.91	21.83	8.91
23.67	19.33	24.67	38.15	53.95	27.66	24.33	20.73	127.67	105.92	45.08	65.69	16.76	6.58
19.24	17.95	21.22	40.85	55.76	24.74	21.42	16.32	120.03	102.93	47.95	68.68	18.06	5.5
24.03	19.5	23.68	37.54	57.67	29.33	23.08	19.8	120.91	106.87	47.16	66.61	20.76	7.7
23.64	21.06	25.03	42.63	57.03	28.82	23.68	21.43	136.02	119.36	50.66	64.84	21.98	6.17
23.7	19.93	22.85	38.42	51.27	26.68	22.02	18.45	116.97	100.48	43.5	58.66	17.93	6.75
23.35	19.31	22.47	39.85	56.86	27.41	19.06	18.66	135.59	105.54	47.35	67.79	22.56	8.69
22.49	19.5	22.25	38.8	52.34	26.78	22.61	20.01	119.08	99.74	42.4	58.66	20.24	7.64
23.95	19.09	24.74	33.55	46.21	26.97	21.18	21.45	104.98	91.41	44	62.25	21.31	7.53
23.7	20.6	23.31	40.13	50.83	29.3	25.49	21.76	134.99	114.58	49.73	68.02	20.49	6.81
19.96	18.47	22.08	33.54	49.1	25.99	19.14	18.64	127.02	106.26	43.2	68.99	17.89	9.34
23.13	17.75	22.8	40.97	62.22	28.5	19.62	18.38	126.34	105.46	53.53	71.72	20.02	8.63
18.76	18.3	22.26	36.75	54.2	26.31	22.28	20	136.66	118.31	49.24	72.52	21.39	8.47
22.72	18.98	22.26	35.84	53.09	29.73	23.49	20.22	143.66	121.37	47	73.69	20.75	8.69
21.45	18.04	19.15	29.6	52.64	25.52	19.07	18.52	125.93	105.24	49.05	65.33	12.39	8.24
21.3	16.62	22.41	37.41	55.41	25.69	19.19	17.45	126.59	107.6	51.19	70.52	17.02	9.38
24.52	20.06	26.25	38.06	63.63	30.84	24.83	24.48	146.68	120.18	52.89	74.43	19.32	7.8

21.45	18.3	23.26	34.7	57.05	26.31	21.94	22.88	134.6	111.3	45.33	68.83	19.09	6.72
21.45	18.28	22.26	35.18	55.69	27.93	20.39	20.2	128.22	112.85	50.29	65.71	21.21	6.51
21.45	13.3	22.26	28.14	44.05	26.31	16.76	15.85	102.27	89.04	40.2	55.76	14.51	7.37
21.45	21.73	22.26	38.02	56.63	26.31	21.16	22.07	130.5	115.12	53.72	71.01	20.01	9.43
27.86	18.55	22.26	36.76	57.59	21.31	22.67	22.77	144.55	128.14	53.64	71.38	20.68	8.38
21.45	18.3	22.26	36.76	53.52	26.31	22.65	19.19	143.99	118.03	49.18	69.9	16.74	8.61
21.45	15.93	18.87	35.4	55.95	26.31	21.77	20.05	135.42	113.79	48.94	66.73	21.58	11
21.45	15.54	22.26	36.76	53.52	26.31	21.02	19.31	120.1	108.85	45.51	64.43	18.95	6.53
21.45	18.3	23.24	36.76	52.55	23.42	20	22.23	129.89	109.26	46.98	69.32	17.58	10.15
22.52	16.37	22.26	36.76	57.06	26.31	20.31	19.42	135.54	110.63	50.01	68.59	18.48	7.98
21.46	18.3	22.26	38.1	56.86	30.88	22.01	23.4	130.48	109.2	54.43	67.4	21.37	7.72
21.16	16.58	22.61	37.89	56.27	27.71	18.33	20.79	125.46	100.99	45.53	67.09	16.32	9.47
22.34	19.05	20.67	36.76	61.57	30.08	23.88	18.76	135.57	120.38	56	67.76	20.78	9.08
20.47	17.46	24.67	39.86	61.55	27.8	20.85	18.67	133.34	109.97	53.76	72.2	17.9	8.24
20.6	20.46	22.63	37.19	53.62	24.75	20.13	18.61	123.7	107.93	48.26	67.61	16.78	8.19
21.12	19.13	23.88	33.83	51.74	26.24	19.62	20.6	120.79	100.83	45.84	60.01	29.88	10.76
24.23	21.89	26.3	35.91	48.97	29.01	22.91	23.3	122.81	98.68	48.55	57.72	27.51	5.97
23.47	20.62	24.48	36.02	52.1	28.46	20.59	24.5	121.65	100.8	49.79	66.66	23.75	6.69
22.49	20.53	26.26	35.37	52.4	26.24	23.61	25.06	123.29	99.73	25.15	60.82	24.25	7.85
21.59	21.59	22.97	39.2	55.86	26.31	23.67	20.37	131.23	102	53.7	65.06	19.74	8.89
20.17	18.43	19.72	39.07	55.4	26.45	19.46	14.96	127.02	102.66	53.13	74.3	15.03	6.69
25.1	19.69	20.68	41.84	58.25	30.45	26.71	21.76	135.21	110.02	52.84	82.5	17.13	10.29
19.11	22.71	23.53	36.19	52.82	27.79	23	19.31	127.02	106.26	48.32	72.12	19.97	8.11
19.95	17.83	19.89	31.53	52.51	23.25	20.69	17.6	117.27	93.74	45.57	60.34	19.66	7.76
23.46	23.43	25.55	38.22	45.16	25.73	20.56	20.83	122.19	95.19	42.76	61.7	23.22	8.57
22.88	18.09	21.38	36.76	46.43	27.25	21.02	25.19	109.79	100.06	48.01	59.41	18.88	7.7
21.45	18.3	23.01	36.12	56.49	25.91	21.49	19.24	128.36	102.89	52.58	74.07	18.65	10.45
24.69	21.64	27.23	37.56	48.71	29.56	23.62	22.92	119.05	102.56	51.35	63.14	22.63	6.84
26.8	21.37	27.82	36.34	52.06	29.67	24.73	22.66	121.46	97.06	49.44	56.67	22.28	6.95
17.92	15.56	12.95	32.99	51.41	NA	11.09	13.89	128.64	108.53	50.48	59.47	21.71	4.78
12.74	17.14	NA	32.62	55.55	25.1	11.93	17.82	127.88	108.28	47.25	63.33	16.85	5.47
13.47	10.24	10.61	37.52	53.34	20.28	10.98	12.49	124.32	109.24	NA	NA	18.4	5.76

15.97	16.47	17.52	NA	58.96	NA	NA	NA	124.17	102.82	NA	NA	18.07	5.64
17.51	14.42	13.28	38.66	57.68	26.61	15.93	15	130.98	105.92	56.65	60.69	21.82	6.36
13.77	14.48	16.19	35.48	53.41	23.35	16.65	14.85	139.79	111.41	54.48	59.97	19.88	6.52
NA	NA	14.58	35.04	57.84	NA	NA	15.27	127.86	110.65	53.21	69.96	NA	NA
20.9	22.57	18.87	33.48	55.28	22.57	17.46	17.26	121.13	112.07	NA	69.02	21.43	6.85
23.86	14.55	16.44	NA	58.61	27.74	17.17	17.77	131.79	108.51	54.29	75.54	24.86	6.71
NA	16.68	17.07	38.41	52.6	NA	20.78	18.04	129.48	105.19	49	71.39	23.33	7.96
17.78	16.21	16.21	36.7	56.9	NA	20.92	16.69	129.08	108.31	53.52	75.53	16.62	5.84
NA	NA	21.58	35.53	58.6	25.82	19.54	16.47	128.24	108.07	52.77	68.2	20.8	7.36
16.84	15.96	20.44	37.69	55.53	20.74	19.83	16.51	126.77	110.05	52.94	64.97	20.98	5.55
NA	NA	16.01	34.51	52.87	NA	NA	NA	121.46	105.76	44.84	69.63	22.8	7.07
19.75	18.16	20.77	37.38	57.28	NA	20.74	16.92	125.28	104.14	53.58	76.66	21.64	7.55
21.64	16.81	21.19	37.45	54.48	NA	17.01	15.53	121.27	109.71	50.59	70.6	20.56	6.4
18.66	16.45	16.12	33.3	53.28	24.8	18.76	10.5	120.89	107.68	42.31	63.96	17.91	6.36
18.19	19.03	17.09	31.63	47.25	23.21	18.96	19.99	125.91	107.89	45.61	64	20.95	6.89
NA	15.43	15.01	29.84	55.96	22.96	16.01	14.28	122.84	108.94	NA	67.84	21.12	5.45
NA	NA	NA	NA	53.54	NA	13.42	13.84	124.01	108.4	48.04	69.64	20.4	5.48
18.46	16.23	18.38	NA	53.65	24.47	13.9	17.7	126.12	111.93	52.7	67.71	21.64	6.52
NA	18.92	18.53	NA	50.94	22.01	17.62	16.72	123.59	110.44	49.8	70.44	19.41	6.77
15.98	15	18.08	NA	57.13	NA	21.92	19.85	127.47	106.08	49.92	68.91	17.52	5.49
20.67	NA	17.33	NA	55.54	27.92	18.45	17.02	125.35	103.75	46.76	61.61	17.33	6.4
17.16	13.5	16.73	39.09	58.57	NA	17.09	14.57	124.6	102.71	50.61	71.02	23.21	7.44
12.34	21.62	14.86	41.49	56.09	31.23	15.81	14.49	124.16	105.97	50.76	65.51	18.19	6.92
19.54	15.29	20.4	36.45	53.9	26.82	20.18	14.77	130.37	106.27	52.59	68.82	19.72	7.1
19.71	16.92	16.73	32.39	55.39	28.41	22.45	18.77	123.81	112.09	53.24	68.84	19.51	7.1
18.66	15.51	14.7	NA	58.65	29.06	22.54	15.98	135.11	108.68	49.34	68.09	19.88	7.27
19.41	12.72	17.57	35.71	52.33	25.89	16.92	15.62	126.06	105.59	45.8	65.89	16.12	7.09
20.54	17.74	18.69	37.27	52.23	27.43	18.46	16.31	135.01	113.21	45.59	66.81	17.38	7.24
NA	18.15	14.66	40.12	54.82	NA	18.25	16.53	132.62	104.77	48.38	71.04	19.76	7.75
20.47	14	16.78	NA	NA	NA	13.06	15.56	128.33	113.07	52.77	71.83	19.48	5.94
13.88	12.64	15.03	32.64	56.58	23.83	16.44	17.36	121.42	103.08	46.9	65.74	17.24	8.68
22.87	14.9	17.46	33.47	60.07	26.67	20.79	22.98	128.94	107.85	50.94	69.9	17.08	5.44

17.32	14.49	18.31	35.95	51.96	31.93	18.7	18.38	126.74	107.82	54.4	74.05	18.83	8.06
18.6	19.48	21.38	37.07	54.42	24.95	19.2	20.48	119.13	107.04	50.64	70.49	19.38	6.4
NA	14.23	18.16	NA	56.39	NA	NA	15.15	131.11	112.72	56.48	73.41	20.47	5.8
22.93	18.56	25.96	38.72	59.83	23.61	19.34	21.95	124.69	115.85	52.42	64.11	15.22	4.76
15.72	15.81	NA	36.73	57	24.86	22.26	20.62	116.94	99.54	50.97	68.48	18.68	6.61
15.56	16.2	16.23	34.12	56.59	21.67	15.08	11.99	127.91	103.41	48.42	66.73	16.76	6.62
15.42	15.89	NA	38.18	52.44	30.11	20.66	18.07	127.98	108.07	44.21	75.24	16.63	7.81
17.79	15.36	17.84	43.08	58.5	25.1	14.86	17.67	134.15	106.85	48.85	74.26	19.12	6.37
22	18.66	21.47	34.76	56.66	29.93	23.04	16.63	118.26	104.81	46.12	67.85	19.83	8.4
23.61	16.96	21.31	37.15	52.31	28.42	23.44	17.62	123.73	102.9	50.93	69.4	19.9	6.49
NA	14.93	NA	33.84	56.4	29.1	18.73	14.25	121.82	100.34	56.04	68.19	16.54	9.19
23.38	17.2	23.55	NA	53.97	26.4	22.35	19.77	128.43	101.29	NA	68.91	18.51	8.42
19.4	17.48	16.98	38.78	57.04	23.72	16.76	15.89	133.08	115.41	51.36	69.09	16.79	8.25
21	17.78	17.14	39.99	63.58	23.6	18.6	16.92	135.16	106.41	52.08	76.73	18.46	6.91
21.68	15.91	19.6	35.68	55.7	25.16	15.88	16.3	137.67	114.19	51.17	70.64	25.67	7.46
17.15	16.49	16.47	39.85	58.48	21.79	13.59	18.95	125.07	113.15	49.1	66.61	23.06	7.78
17.8	16.65	22	31.02	55.61	27.38	17	16.56	115.32	106.23	47.85	NA	16.71	6.23
NA	NA	21.48	40.14	60.37	NA	18.93	18.48	128.91	108.76	48.66	67.4	22.11	8.89
19.31	16.5	19.39	38.62	60.98	NA	20.03	15.51	134.49	99.16	46.52	66.01	18.93	6.21
21	NA	20.96	35.91	51.44	20.53	NA	16.92	121.09	108.58	48.74	58.34	16.39	6.81
18.51	19.82	NA	34.7	51.05	25.22	20.54	17.8	130.14	109.42	53.88	72.86	19.23	6.63
NA	NA	NA	NA	54.81	NA	18.93	18.06	133.46	111.75	47.97	67.82	17	8.98
14.34	14.17	16.72	40.27	57.1	27.99	17.99	19.38	121.13	109.87	51.39	68.08	17.39	7.22
18.26	18.54	13.53	31.75	53.5	21.32	18.59	18.06	122.58	105.6	45.66	70.09	19.76	6.36
NA	NA	17.48	38.33	65.21	NA	19.26	18.28	123.29	99.44	52.52	74.16	18.43	8.24
16.69	16.96	17.96	31.64	54.1	24.11	18.82	16.92	123.66	105.08	48.46	71.51	19.21	6.46
19.74	18	18.63	35.86	53.62	21.61	18.35	16.45	131.06	102.05	45.66	68.11	21.51	6.66
20.52	NA	18.39	40.35	59.9	25.44	19.58	13.26	128.88	111.97	NA	70.83	19.04	7.51
15.17	13.52	17.71	34.41	60.75	24.04	20.6	15.53	130.68	109.82	49.92	66.53	17.77	6.35
13.44	16.5	20	38	65.27	20.78	17.91	19.85	126.01	108.71	51.67	70.52	20.06	6.74
18.5	16.55	20.22	34.14	66.03	22.61	20.71	15.47	131.87	112.83	51.08	71.76	19.83	7.89
19.72	16.5	23.46	37.47	54.86	21.47	21.23	18.32	126.77	113.47	46.91	71.1	19.83	9.7

14.82	11	17.36	NA	57.65	21.56	21.84	15.3	131.96	107.82	46.8	67.71	17.47	6.96
NA	NA	21.24	NA	58.59	25.17	20.58	16.68	134.33	109.17	48.28	64.85	17.87	6.21
16.94	15.1	NA	36.18	63.05	20.24	17.92	17.82	130.23	114.63	52.52	69.71	17.16	6.25
13.59	NA	17.65	40.34	60.85	NA	15.84	16.47	136.73	112.36	51.52	72.91	19.38	8.08
12.28	15.21	24.62	NA	55.2	NA	12.8	14.96	127.39	109.48	49.38	69.68	18.41	8.1
21.47	13.59	18.83	34.58	60.15	NA	17.71	22.89	134.23	114.81	41.06	68.74	18.26	10
NA	NA	NA	NA	58.37	25.25	17.27	15.54	138.14	111.84	47.97	71.51	19.07	7.11
18.01	15.59	19.45	NA	54.03	NA	15.67	14.26	126.84	114.96	50.6	68.28	20.79	6.17
NA	NA	NA	NA	57.3	24.31	21.73	16.01	130.56	110.85	51.82	74.32	19.51	8.1
NA	15.68	16.27	34.34	54.51	24.51	18.8	17.8	130.7	110.54	42.5	66.39	15.3	5.72
17.2	15.75	20.11	NA	55.44	NA	15.89	15.32	121.49	112.91	48.4	58.37	21.4	9.72
15.41	NA	23.95	41.02	57.2	32.1	16.98	17.17	126.84	110.89	48.9	71.26	22.2	7.98
NA	NA	19.07	43	55.25	NA	17.48	19.64	122.07	110.46	39.31	68.11	19.36	8.89
17.49	17.77	19.27	33.9	54.79	25.44	19.53	17.14	124.46	106.61	47.96	61.44	15.74	7.05
17.64	17.94	16.86	35.49	54.65	20.45	20.68	14.9	114.74	102.33	49.84	68.33	17.68	8.43
20.16	15.14	17.31	37.26	52.09	24.42	20.57	18.5	131.92	113.58	50.32	69.38	18.39	6.4
21.4	18.18	20.7	37.63	53.31	27.4	16.79	18.72	130.62	110.48	54.87	70.84	18.55	8.73
15.09	10.53	21	42.58	55.29	22.63	16.01	16.55	129.37	112.15	46.87	71.48	17.85	5.55
19.06	11.59	NA	NA	55.7	22.06	18.72	10.73	130.51	118.1	48.39	67.25	16.87	8.12
NA	NA	16.38	NA	61.37	NA	20.7	14.26	128.7	112.4	48.62	62.51	17.82	8.01
21.26	14.75	17.13	36.91	54.81	27.31	19.21	14.8	125.36	114.35	52.24	66.53	19.17	6.88
18.57	18.58	16.47	34.04	52.28	26.38	19.29	15.39	132.93	109.45	46.32	57.52	19.73	7.36
14.32	NA	16.26	38.18	51.86	20.66	16.67	14.54	122.52	98.61	46.44	63.64	16.75	9.65
18.6	12.37	18.68	31.16	57.19	22.8	22.1	16.3	119.32	110.81	56.23	69.87	20.17	9.91
18.52	NA	22.49	30.78	58.8	24.43	15.1	16.07	128.04	103.59	47.01	69.66	18.97	8.27
19.29	16.99	18.72	35.22	55.11	24.12	16.87	17.31	123.06	104.05	51.09	60.24	19.19	7.95
20.61	15.91	20.02	33.4	61.03	25.71	17.63	16.94	126.04	110.29	47.09	70.49	19.7	6.95
18.75	14.76	19.9	36.04	60.36	26.55	17.44	15.39	126.67	115.96	49.69	64.81	18.83	7.41
24.25	20.21	19.71	31.5	56.06	24.84	19.28	17.24	132.35	111.26	48.32	69.29	20.97	6.88
18.57	15.21	18.3	37.86	58.39	29.2	19.74	18.02	128.26	112.18	53.35	70.08	20.34	7.04
17.85	17.67	18.74	35.56	53.56	22.98	22.2	16.83	127.06	101.3	50.35	73.18	24.55	8.57
20.48	15.37	18.19	32.37	52.02	28.33	18.9	18.45	124.2	110.06	54.51	71.11	17.79	7.5

16.45	16.75	16.16	35.55	53.75	25.31	17.25	17.31	125.07	114.11	50.05	65.53	22.92	9.59
18.9	15.98	NA	35.99	57.89	26.64	14.37	16.56	130.38	108.81	54.61	70.4	17.24	6.71
18.52	14.25	21.96	NA	56.13	26.85	15.48	14.58	128.89	105.75	50.24	65.63	25.97	9.04
21.72	18.52	18.36	34.37	55.1	27.72	20.62	16.32	123.08	110.67	42.62	66.52	19.36	7.4
16.4	10.84	16.43	25.7	53.13	22.95	15.1	17.43	134.12	113.12	46.66	72.82	24.84	9.99
19.33	15.34	18.11	39.77	53.62	27.45	14.24	15.1	138.88	118.34	45.25	66.43	17.46	5.96
19.17	16.13	20.32	37.56	58.45	18.97	19.56	18.5	132.63	111.55	57.32	65.85	16.14	6.01
NA	NA	16.63	NA	56.05	NA	19.58	16.89	130.7	115.09	51.01	70.89	18.59	6.52
17.92	16.87	17.56	NA	52.3	NA	16.8	15.68	131.23	106.87	46.16	72.29	17.3	7.31
19.47	16.4	19.62	37.75	59.22	31.44	14.72	18.57	125.7	108.98	48.72	75.91	17.28	7.04
NA	NA	19.52	40.02	60.06	NA	NA	17.11	132.65	108.68	49.27	71.18	19.58	8.86
20.38	16.79	20.33	40.77	54.93	29.83	16.03	15.95	132.98	106.14	49.12	67.04	20.55	5.66
20.17	16.1	18.08	37.71	59.12	20.91	19.28	15.98	123.95	117.7	38.35	70.84	20.19	6.39
18.46	15.91	18.62	NA	57.52	NA	15.88	15.47	133.01	108.31	48.47	73.56	18.68	9.55
17.69	NA	NA	NA	68.74	18.92	18.6	18.92	126.91	109.99	54.92	76.1	16.24	7.31
17.68	16.87	19.4	37.71	54.64	22.39	17.79	17.49	135.33	114.31	52.41	66.57	18.08	5.85
21.48	18.72	NA	NA	57.63	NA	20.01	14.92	136.3	115.05	56.41	75.91	19.36	7.25
19.43	21.05	18.15	36.32	61.12	21.64	16.37	14.48	125.99	109.48	42.3	62.25	18.62	8.23
17.93	18.18	20.42	39.93	55.5	29.95	21.84	19.95	139.78	117.96	55.66	73.2	16.81	9.82
17.71	17.88	19.58	37.65	58.47	29.46	20.54	17.07	134.1	113.28	56.14	71.39	16.92	10.5
17.27	13.69	18.1	NA	61.69	NA	17.68	19.13	136.34	115.27	52.43	74.88	16.46	6.15
16.64	14.72	20.54	37.83	61.15	23.37	17.18	19.58	130.8	116.39	53.6	72.72	20.5	9.96
NA	14.49	NA	35.06	NA	18.84	15.15	17.17	127.7	110.59	47.48	67.44	18.64	5.76
17.68	17.74	19.36	34.46	59.26	NA	18.55	19.11	136.6	110.27	45.41	66.3	19.17	8.48
22.07	15.1	20.17	34.7	60.25	29.22	18.43	14.81	137.67	110.35	49.11	66.24	20.47	8.39
16.05	14.79	19.01	30.63	52.88	22.1	16.32	15.5	129.36	106.96	NA	60.47	21.17	7.3
16.96	20.64	22.32	44.78	56.53	26.28	16.84	17.13	123.87	113.35	48.65	NA	19.06	6.96
14.14	15.13	19.4	28.6	51.43	32.94	19.34	17.53	127.72	109.49	43.79	68.88	17.04	6.77
16.11	14.32	20.4	26.09	53.43	26.89	22.73	20.58	135.21	113.41	42.73	NA	19.07	5.92
21.25	18.81	25.66	38	53.52	27.89	19.95	19.99	126.99	104.93	NA	73.81	17.57	7.95
16.26	13.76	18.92	NA	62.19	25.13	17.14	19.06	130.56	105.86	NA	NA	17.54	7.96
16.11	17.43	17.86	34.64	58.49	27.65	17	19.75	125.6	106.44	NA	62.3	17.17	6.76

20	16.35	22.12	33.39	NA	29.09	19.07	15.24	131.79	110.08	NA	NA	17.5	5.67
16.92	19.99	14.68	NA	NA	30.21	20.27	19.36	126.48	104.48	48.72	62.9	17	9.3
22.08	20.49	21.01	39.12	NA	28.63	20.26	16.37	128.1	102.27	40.77	63.09	NA	NA
14.65	14.4	19.24	NA	NA	NA	20.27	16.93	125.1	102.33	53.35	66.44	20.92	6.12
21.36	16.03	15.92	38.04	59.33	23.75	14.62	15.01	127.29	102.08	53.17	NA	17.18	7.81
20.06	14.05	18.18	40.77	52.89	28.96	21.56	21.69	127.14	111.54	48.93	NA	17.26	5.17
19.65	15.95	15.79	NA	NA	29.44	17.77	16.72	131.38	103.59	NA	57.21	17.81	7.92
20.58	16.4	19.95	35.85	56.14	28.47	20.36	20.84	126.67	107.85	48.87	58.41	21.61	7.94
14.97	17.07	19.07	NA	NA	26.23	17.35	19.75	129.29	106.01	52.65	72.08	18.25	5.67
14.3	13.08	15.92	36.49	54.33	22.7	18.94	14.5	121.71	104.02	45.48	73.61	14.85	7.2
19.95	19.09	22.19	NA	NA	27.79	17.7	21.11	130.64	105.96	49.06	NA	15.27	7.44
12.83	13.34	17	38.49	NA	26.88	20.56	19.56	129.57	112.19	NA	60.25	19.12	6.39
20.56	18.12	18.62	34.66	50.25	25.55	19.59	22.5	129.89	106.69	44.53	75.25	23.83	7.46
19.24	12.12	20.39	33.38	62.06	28.99	21.29	17.8	135.42	110.44	NA	75.63	23.62	7.17
16.34	16.08	18.14	41.27	55.54	29.06	18.58	16.13	136.24	105.22	53.36	65.73	19.44	7.75
18.87	15.65	20.08	NA	NA	24.63	18.81	14.97	124.37	110.22	NA	NA	16.22	7.76
18.5	18.64	16.82	35.71	60.35	23.64	21.22	19.04	115.58	108.55	NA	79.27	22.45	9.94
19.92	16.06	15.92	31.87	58.33	22.77	17.78	16.03	124.75	106.16	NA	65.99	21.09	6.79
19.3	19.6	14.48	NA	NA	26.96	22.85	23.35	123.44	96.58	40.71	NA	14.94	6.22
17.94	16.43	21.17	38.52	61.04	27.92	20.62	17.54	127.44	103.65	55.78	68.57	21.56	6.3
18.86	18.81	22.19	47.74	54.76	36.78	18.67	21.62	124.39	101.47	56.98	NA	22.44	9.25
17.6	15.83	16.52	30.09	68.35	19.13	18.86	12.98	118.84	103.09	NA	NA	20.93	10.24
25.27	21.19	17.92	36.95	NA	NA	18.81	20.69	131.32	105.76	51.96	NA	20.33	8.41
21.91	15.66	20.9	34.63	NA	29.3	24.68	17.43	123.95	113.16	42.84	65.96	19.37	5.68
19.88	17.38	21.09	NA	NA	26.14	21.68	17.31	129.28	101.64	43.36	NA	22.32	7.94
NA	NA	NA	NA	NA	33.49	21.99	19.94	125.19	112.42	43.9	NA	19.01	9.12
16.22	16.99	17.5	40.56	55.18	24.69	22.95	20.2	125.67	107.77	44.61	NA	14.99	7.2
15.92	14.14	17.68	37.85	60.85	35.68	23.03	15.47	124.08	109.22	38.06	68.84	14.79	7.65
6.41	6.27	5.95	11.62	72.79	10.71	10.57	7.81	112.38	84.41	18.38	36.01	24.71	2.01
8.09	7.01	9.23	17.26	67.67	11.84	10.2	7.75	98.63	85.76	17.29	33.55	32.05	2.2
7.33	4.4	8.24	16.49	69.77	12.64	8.69	10.45	113.18	93.29	15.68	36.67	26.72	2.32
10.31	6.78	12.13	16.06	66.76	11.05	10.19	10.19	105.55	86	20.18	37.09	24.68	3.09

7.49	7.04	6.26	14.62	79.76	13.77	11.56	9.36	106.67	91.64	16.05	29.33	21.69	2.51
9.66	6.64	9.49	15.09	68.38	14.62	12.42	12.73	106.58	86.17	18.44	36.59	24.08	2.67
11.6	6.75	12.05	18.67	76.77	13.88	14.08	13.09	106	86.33	12.46	32.21	33.14	3.22
6.06	6.45	8.07	10.64	66.68	9.55	9.31	8.01	95.1	94.51	16.98	29.06	23.78	1.57
15.07	8.91	18.06	26.29	77.34	18.12	15.84	13.48	134.73	99.73	31.05	48.49	5.18	11.24
15.37	11.02	11.68	21.17	53.84	18.23	17.58	10.64	121.98	102.72	37.32	47.89	4.36	11.66
7.68	7.27	9.39	20.06	78.32	12.7	12.4	9.8	123.64	105.95	37.33	46.47	3.99	9.95
11.58	9.26	11.49	23.16	80.37	14.21	14.4	13.71	131.98	106.34	35.83	49.13	3.88	7.68
7	7.07	8.35	10.65	49.56	15.05	12.01	12.59	117.9	70.78	34.12	56.7	4.13	4.29
8.77	7.39	10.01	11.66	54.5	20.26	12.48	9.12	109.79	66.55	29.34	60.17	NA	NA
8.61	8.98	13.42	10.44	57.14	NA	8.68	11.61	107.55	69.91	23.02	53.58	5.2	5.29
9.63	8.3	12.49	14.69	58.35	NA	11.66	NA	114.72	63.84	35.05	58.25	4.46	5.08
9.32	6.63	13.05	11.65	61.25	12.13	12.72	10.2	118.57	70	35.6	60.94	5.07	5.65
8.3	8.3	7.67	10.46	52.18	14.81	12.05	11.71	106.37	67.08	26.97	60.98	3.77	5.07
NA	10.99	10.99	13.91	56.73	13.89	12.4	11.61	115.07	69.94	32.55	55.98	3.49	5.26
6.91	6.77	9.27	8.61	47.8	11.91	7.22	10.01	113.68	63.82	26.87	49.74	3.59	5.75
7.24	6.33	7.96	8.79	44.23	NA	9.42	9.42	109	63.22	25.97	48.78	4.22	4.78
7.58	7.74	8.58	12.05	54.75	14.19	9.83	10.77	111.42	59.68	26.09	47.2	5.27	5.44
8.04	1.85	5.65	96.43	279.67	99.77	8.66	8.66	104.35	55.98	8.16	NA	20.78	2.56
7.33	5.02	8.16	121.84	295.33	104.35	14.09	9.24	113.06	59.16	11.91	57.45	17	2.68
7.99	6.53	11.11	126.01	299.45	121.39	9.94	10.97	122.79	54.5	10.98	69.18	29.37	3.18
5.68	6.07	6.84	124	252.57	NA	13.81	NA	110.12	56.78	13.43	81.55	27.73	3.96
4.07	7.33	6.01	126.76	331.58	106.24	9.43	8.6	112.82	55.13	11.67	79.23	25.12	4.21
8.1	4.77	7.65	117.94	345.3	NA	10.5	NA	125.65	55.8	11.81	87.75	24.26	3.97
7.85	5.69	4.83	102.39	312.02	101.25	7.5	7.19	119.82	54.83	11.83	NA	26.48	5.05
8.68	5.03	7.2	122.06	NA	94.06	11.38	7.5	115.2	57.74	6.41	NA	23.58	3.5
10.16	5.78	8.18	108	296.44	96.15	8.91	7.87	124.17	55.93	9.85	84.2	31.13	NA
5.41	4.94	5.55	104.1	314.2	94.63	9.48	6.53	110.15	57.83	5.56	74.37	NA	NA
9.28	7.51	7.45	37.37	56.11	24.32	18.1	11.07	109.35	104.03	37.62	56.77	42.97	8.64
16.92	11.7	15.63	44.88	58.29	33.8	17.17	15.14	123.06	106.76	40.32	60.51	31.57	9.58
15.28	9.22	13.27	42.78	56.86	31.16	17.05	13.14	107.62	101.74	42.01	60.59	34.13	6.15
15.51	8	13	42.73	59.98	33.19	17.1	12.83	117.19	105.28	44.49	55.66	32.23	9.05

14.5	11.1	13.38	41.66	55.41	32.07	20.15	13.3	115.13	96.52	38	59.91	37.71	8.51
6.13	5.31	9.48	42.7	59.08	31.29	12.44	9.36	109.92	96.43	45.83	60.51	NA	NA
16.11	8.67	9.08	38.03	58.35	22.82	12.09	9.58	112.75	94.83	34.81	59.68	33.72	4.04
9.34	9.09	10.07	34.48	51.81	25.3	11.02	8.37	115.22	93.67	34.98	53.93	34.41	4.11
10.37	7.52	6.97	31.76	61.04	24.37	8.86	5.54	94.07	94.05	36.48	56.84	33.7	3.54
11.17	8.31	9.22	53.66	50.64	NA	9.39	7.68	106.74	93.2	38.15	60.95	34.72	9.16
8.87	8.22	8.56	27.98	71.91	18.75	10.21	9.36	98.85	83.56	24.72	52.85	16.75	8
10.4	6.01	11.23	45.76	99.19	23.78	11.93	12.5	NA	NA	25.12	70.24	NA	NA
7.8	4.08	11.35	35.52	78.29	NA	11.05	8.52	109.75	93.07	30.67	56.22	NA	NA
7.78	4.39	8.72	35.6	89.99	NA	16.24	11.38	104.53	94.83	27.46	56.32	12.17	6.32
12.26	7.18	10.89	33.51	79.32	NA	8.57	9.22	109.33	93.82	26.08	55.06	17.23	6.57
15.22	6.44	10.15	38.4	86.03	20.28	11.85	7.86	111.14	95.91	27.29	59.25	14.19	13.47
9.5	7.86	8.36	37.28	90.12	20.24	10.19	9.14	110.98	91.48	29.56	61.19	13.07	10.64
9.87	6.36	9.71	38.55	88.54	15.89	8.42	7.56	106.53	89.31	27.11	56.51	13.95	9.2
7.97	6.95	8.57	35.73	84.45	13.9	11.7	8.29	108.45	99.61	27.54	57.99	16.1	13.81
12.2	9.99	17.44	42.74	91.76	NA	12.08	9.87	124.65	96.6	29.56	NA	17.98	10.79
9.63	8.73	10.09	17.71	46.47	9.32	10.85	10.21	104.74	79.31	22.05	20.94	2.33	7.23
8.88	7.44	8.35	15.62	57.78	8.52	9.29	6.89	113.14	92.99	29.27	26.69	3.4	9.94
8.87	7.04	11.78	16.6	53.53	14.28	12.69	9.92	111.56	91.36	26.29	24.48	4.01	12.13
8.66	10.62	6.53	20.8	57.45	14.49	9.34	8.13	102.81	86.64	23.95	24.98	NA	NA
9.13	10.58	8.86	26.77	51.55	8.67	10.97	11.66	112.45	85.98	28.55	25.02	3.98	9.87
7.81	8.14	9.48	16.19	55.53	10.78	10.58	9.92	114.45	86.19	26.59	23.86	3.77	11.95
85.58	42	84.34	75.62	89.4	91.19	61.4	48.72	119.45	91.14	73.29	87.77	23.18	5.61
92.08	40.11	84.88	77.31	93.77	98.35	65.46	51.66	118.47	92.25	71.51	82.87	21.92	6.28
81.32	42.06	94.86	68.75	83.91	83.65	65.89	64.82	121.96	81.98	71.69	NA	23.87	6.79
91.48	NA	89.48	69.41	91.77	87.46	69.8	78.2	113.52	94.3	75.08	81.4	19.63	7.2
72.01	34.84	74.97	77.96	86.89	88.62	69.11	63.37	118.6	103.39	65.23	89.53	21.71	5.4
58.78	35.32	NA	82.28	87.58	102.32	62.97	67.35	113.8	97.44	73.22	86.14	16.83	5.73
79.1	27.31	91.48	81.24	82.21	89.34	52.1	65.01	114.59	102.47	73.37	88.07	22.35	5.52
66.4	31.9	71.6	NA	NA	87.92	58.09	54.56	121.15	96.78	61.41	83.03	20.71	4.88
56.02	24.72	64.4	NA	63.96	70.55	55.45	44.77	111.23	89.23	56.26	72.63	14.68	8.39
77.2	34.2	82.78	84.19	85.2	102.82	70.28	75.46	121.2	96.57	91.87	70.98	NA	NA

12.84	8.66	9.82	32.39	82.44	20.26	14.93	12.66	115.38	103.2	39.97	49.56	20.95	9.33
8.46	7.59	14.02	25.28	73.35	25.55	15.03	14.92	104.52	112.2	29.26	45.53	21.53	11.61
10.23	9.77	12.58	25.63	71.41	19.59	10.66	11.92	112.39	119.09	29.36	40.71	18.88	11.93
11.12	11.59	13.45	26.53	73.23	19.23	15.11	14.15	117.3	120.6	29.63	40.85	19.07	11.63
11.99	9.67	11.97	25.64	74.57	23.35	13.89	12.34	112.75	115.74	33.62	42.2	20.02	11.61
10.61	10.44	15.89	24.35	70.85	23.06	11.01	12.49	114.84	116.81	27.02	44.09	20.96	8.95
12.23	10.69	12.52	33.61	68.22	28.73	12.42	11.44	106.66	121.06	32.2	42.93	20.3	11.76
13.94	7.83	11.98	26.3	78.12	23.01	9.43	14.7	113.9	121.55	30.69	50.31	23.98	13.64
81.01	26.03	68.92	69.2	80.18	75.11	40.8	59.68	121.65	92.52	57.94	77.2	NA	NA
56.05	22.54	NA	72.39	82.15	68.72	50.91	54.03	116.22	88	55.17	NA	NA	NA
61.18	22.12	63.94	65.85	78.87	71.88	47.67	50.29	112.42	84.95	57.42	53.9	NA	NA
63.01	23.4	66.76	63.06	77.89	68.34	54.15	47.31	130.93	91.71	59.49	81.06	NA	NA
63.24	NA	67.65	57.13	76.5	71.31	55.72	52.98	122.12	94.46	51.81	77.54	19.48	4.08
57.54	26.32	59.62	64.55	NA	NA	NA	58.35	127.79	89.12	72.16	75.49	19.92	4.53
65.35	28.62	69.84	62.86	84.08	74.04	55.06	54.2	117.33	86.72	70.71	62.86	23.71	4.19
72.29	30.02	79.35	75.64	99.94	85.73	53.2	NA	133.89	91.14	77.14	84.12	25.28	3.89
76.97	NA	NA	NA	NA	NA	60.7	61.82	130.98	95.61	67.77	83.8	19.83	6.11
11.58	5.29	8.69	NA	203.58	63.78	16.36	10.26	110.34	72.66	65.29	66.85	13	3.51
16.4	5.28	8.2	86.82	178.28	81.23	20.71	11.82	118.31	76.68	73.13	NA	16.01	3.04
10.44	9	8.7	89.53	176.1	68.21	13.46	11.6	113.37	73.57	65.59	70.04	14.94	3.74
6.77	NA	NA	96.64	218.09	NA	12.92	13.07	NA	75	NA	NA	18.04	5
10.02	NA	NA	106.26	254.58	85.52	NA	NA	127.72	87.26	66.81	65.16	11.59	2.87
15.85	6.63	8.48	NA	NA	89.43	18.43	13.08	130.96	91.46	84.05	NA	14.26	4.3
9.31	6.91	9.84	127.13	259.3	87.84	17	10.02	119.58	80.35	66.06	75.46	16.91	3.93
10.34	5.02	7.75	NA	NA	NA	13.03	9.61	110.68	77.44	54.4	NA	14.54	3.88
13.29	8.97	9.88	111.26	NA	62.93	11.85	7.65	122.27	91.49	62.69	62.57	NA	NA
10.55	4.38	7.97	NA	131.07	NA	17.82	8.49	125.49	94.86	NA	71.53	10.76	12.35
12.1	6.9	8.6	NA	NA	45.67	17.5	9.4	NA	NA	NA	69.99	11.33	13.4
10.92	6.57	8.18	57.22	157.48	60.53	19.94	NA	150.75	117.39	61.15	83.62	NA	NA
13.32	4.99	9.02	54.55	142.09	52.88	19.05	9.96	137.05	94.9	53.24	77.83	NA	NA
11.19	5.54	6.57	46.69	116.56	NA	19.81	9.24	129.69	89.79	50.19	66.43	13.77	13.43
11.28	7.68	8.19	53.02	153.09	51.4	21.28	10.86	136.56	98.74	56.1	69.04	NA	NA

11.94	8.03	9.61	52.53	119.72	44.78	18.82	10.33	120.96	92.99	49.39	67.01	11.9	12.61
10.08	5.15	7.51	51.41	141.24	50.34	18.49	10.03	128.25	98.98	47.66	75.37	NA	NA
8.38	6.33	7.23	50.38	142.39	42.91	21.32	10.68	NA	93.48	44.73	73.4	9.92	10.72
11.36	4.66	7.99	51.95	119.22	45.82	20.53	12.7	120.76	99.81	45.29	72.5	10	10
5.19	5.14	3.94	101.34	235.37	84.85	9.85	8.67	108.72	50.33	48.37	69.89	29.59	5.29
8.3	5.96	6.88	89.88	250.41	82.55	7.45	7.78	108.74	52.37	49.58	67.25	29.96	7.14
6.61	5.38	4.37	106.99	287.74	96.33	7.67	6.33	112.13	50.95	62.99	69.32	33.97	8.9
6.67	7.49	4.56	88.34	235.92	89.58	4.8	5.86	103.25	43.87	50.27	62.33	24.85	4.56
8.71	3.03	8.07	85.11	238.1	92.36	9.71	6.78	NA	NA	49.59	69.69	28.4	5.76
6.19	4.11	5.45	92.16	222.46	81.61	6.34	5.98	99.28	43.52	53.9	60.06	28.52	4.44
6.05	4.41	7.14	94.86	NA	94.09	7.64	8.86	113.73	48.61	61.25	59.67	26.24	5.3
6.19	2.63	6.07	101.28	199.56	85.34	7.6	6.59	102.02	51.31	59.49	70.18	24.36	6.55
8.56	3.86	7.77	93.93	232.26	92.14	13.67	9.14	100.4	47.49	51.8	NA	25.05	4.79
8.03	5.98	6.98	105.22	258.69	101.34	6.76	9.4	113.27	55.4	67.99	65.81	27.09	4.36
5.99	5.83	8	123.47	227.22	78.6	9.81	8.74	121.69	106	90	63.17	19.73	4.12
8.47	4.51	6.76	113.77	246.08	90.23	9.95	7.21	111.96	101.74	77.39	59.69	21.65	3.65
7.16	3.71	7.74	94.75	240.73	66.32	10.43	6.55	120.66	96.39	71.47	73.26	19.61	2.77
6.18	4.95	4.46	108.75	209.4	82.38	10.32	8.47	118.43	89.52	66.26	57.18	14.32	3.61
6.81	5.83	3.79	109.16	231.71	91.13	6.21	6.94	102.66	94.49	78.29	68.88	18.16	2.67
4.4	5.26	5.04	116.76	226.47	83.91	8.47	6.86	119.32	98.25	86.15	66.72	17.4	3.35
6.86	6.94	5.34	116.72	232.95	88.46	9.61	6.86	118.11	91.75	90.02	66.86	18.31	2.63
6.18	8.01	7.21	125.34	250.86	94.73	10.52	11.11	125.09	92.8	91.91	69.05	18.71	3.26
4.47	4.14	5.66	105.02	239.35	88.83	9.05	7.24	125.18	96.53	94.77	62.47	15.61	3.48
4.17	5.58	6.81	NA	221.84	89.93	8.57	8.48	112.88	93.07	77.61	71.4	14.15	3.68
13.22	7.51	8.92	9.64	52.2	23.53	15.35	12.63	99.14	48.18	32.41	66.14	15.99	6.26
18.88	8.74	12.37	11.12	53.21	26.62	10.29	11.34	NA	NA	NA	57.83	11.7	8.03
17.2	10.13	13.32	13.23	49.97	29.14	NA	12.4	99.95	59.09	28.87	55.08	10.9	8.09
17.73	9.01	13.21	13.66	52.47	24.96	13.3	11.28	102.4	54.58	27.96	59.01	12.16	9.96
22.07	8.77	14.05	14.19	52.61	30.92	17.24	13.8	98.49	50.43	38.89	63.22	15.12	8.77
19.12	10.13	12.6	13.2	57.65	NA	NA	14.34	99.28	50.98	35.02	56.41	11.43	6
14.73	9.94	14.23	NA	58.94	27.33	15.89	13.5	92.08	50.81	36.24	62.51	NA	NA
12.28	10.73	11.44	9.91	46.61	NA	NA	NA	88.09	48.65	24.32	47.58	10.09	9.24

14.44	7.57	12.29	10.23	45.1	21.7	14.88	11.43	88.07	45.37	28.32	50.51	14.55	8.92
15.64	8.64	11.22	11.24	51.18	24.46	NA	14.47	91.62	49.79	37.58	60.17	NA	NA
21.29	14.34	13.71	14.25	36.72	25.37	17.5	7.66	81.19	NA	16.69	50	NA	NA
24.22	15.94	15.75	15.81	NA	31.11	19.6	14.59	84.7	40.33	22.33	NA	15.07	1.62
23.07	17.85	17.48	17.65	38.59	33.61	23.84	14.84	81.12	38.85	23.15	42.92	14.72	0.67
23.52	17.85	15.21	16.35	38.38	35.23	22.33	11.54	87.47	42.5	24.99	NA	13.45	0.86
23.17	15.53	15.35	15.77	38.54	32.87	19.8	13.85	84.73	38.93	24.01	47.05	13.7	0.52
24.51	18.32	16.66	16.64	42.85	34.24	21.14	14.47	85.26	42.18	24.08	50.17	13.5	1.63
7.79	5.58	5.76	9.03	46.26	14.12	8.14	7.22	90.63	48.82	21.63	55	7.81	5.7
5.57	5.33	6.27	8.11	49.22	15.49	8.34	6.51	93.59	54.9	20.18	50	6.5	4.17
6.66	3.9	7.05	8.29	45.71	7.38	8.32	6.42	83.32	48.66	17.08	55	7.29	6.32
10.44	5.47	8.73	9.83	54.3	NA	10.29	9.59	99.1	48.33	28.93	60.4	5.45	6.65
12.44	6.39	10.56	10.91	55.67	20.22	10.66	9.3	89.23	60.33	26.43	64.84	8.77	NA
12.44	6.63	8.65	10.42	56.04	24.28	9.82	10.2	95.06	49.95	28.93	66.6	8.45	8.01
7.79	5.58	5.76	9.03	46.26	14.12	8.14	7.22	90.63	48.82	21.63	55	8	6
29.69	14.81	18.75	11.43	43.37	34.01	26.27	14.02	79.47	35.38	25.2	35.85	20.7	7.37
23.36	12.08	11.57	7.35	38.24	29.64	23.75	12.34	83.92	28.04	17.71	32.45	NA	NA
22.64	10.95	9.93	12.54	38.81	26.94	25.59	10.49	78.88	35.52	20.5	30.58	20.7	8.01
24.16	12.73	10.31	9.89	41.81	32.24	24.9	NA	73.51	36.9	22.21	33.49	NA	NA
24.33	12.82	10.3	11.59	40.81	31.18	23.61	11.08	79.82	NA	23.33	38.63	NA	NA
26.98	14.05	12.87	11.78	41.4	34.63	28.38	14.43	89.66	38.96	24.22	34.34	14.2	5.41
24.74	15.47	13.43	14.89	44.94	34.87	25.09	17.8	85.56	36.91	22.98	37.91	17.74	6.8
28.79	16.38	14.79	13.44	45.06	36.3	28.86	14.5	86.33	38.64	25.37	40.44	20.28	6.05
29.42	20.07	13.38	14.13	45.62	34.08	27.1	14.91	83.62	36.82	25.15	37.68	22.32	6.18
17.52	21.23	11.83	10.04	40.47	28.2	22.3	10.93	75.05	36.9	24.01	31.22	19.88	6.56
27.71	15.03	13.76	13.25	38.17	33.47	29.32	14.52	78.47	35.46	23.88	41.28	21.15	9.12
7	8.1	4.98	7.41	55.88	15.09	14.25	8.21	119.9	52.78	26.63	59.51	18.96	1.58
7.83	4.27	7.8	6.21	54.62	14.68	10.93	9.77	117.39	55.1	28.31	54.66	18.31	2.78
7.6	5.28	6.63	7.86	51.11	14.83	NA	6.21	107.73	55.72	24.94	50.3	19.01	3.67
5.88	4.18	NA	6.23	48.68	13.09	NA	10.66	111.88	52.85	20.97	63.67	18.96	2.65
5.91	7.99	7.57	7.3	51.56	12.59	NA	6.47	106.17	48.94	28.42	61.19	20.92	2
7.54	5.31	8.04	8.05	54.66	11.93	11.1	8.72	120.79	51.44	28.88	59.15	NA	NA

7.31	5.02	9.18	8.05	54.72	13.02	NA	9.45	116.34	57.68	30.42	55.44	NA	NA
7.73	5.58	9.89	7.76	49.91	12.91	NA	NA	NA	48.37	27.45	57.49	NA	NA
7.91	4.72	6	8.35	58.08	13.45	10.86	7.83	112.38	54.55	25.96	52.47	14.51	2.12
6.22	6.2	6.75	7.82	55.53	9.58	13.12	7.26	115.86	54.71	30.25	52.41	16.63	2.38
4.83	5.28	6.1	4.61	47.25	9.41	5.55	7.79	96.37	72.16	20.33	53.67	10.62	1.78
6.13	5.74	7.52	7.15	55.07	8.77	7.74	4.72	104.18	79.34	23.09	58.14	11.85	1.95
5.04	5.72	6.56	6.99	52.5	10.52	9.87	6.3	105.53	74.98	22.81	50.73	12.35	2.28
4.95	4.36	6.74	7.1	50.23	10.4	8.07	7.36	96.67	73.77	21.65	53.92	NA	NA
5.68	4.37	5.68	6.58	53.05	10.54	7.36	4.35	102.78	78.51	22.26	56.15	12.23	1.8
5.27	5.15	5.11	6.92	56.63	8.34	4.92	4.69	101.22	76.34	22.58	57.5	13.19	1.89
18.9	6.64	7.99	13.92	59.7	24.14	17.17	11.43	86.92	51.18	NA	73.13	16.79	4.7
18.63	6.79	10.07	10.37	60.25	26.47	18.82	13.24	88.36	46.32	35.03	69.46	14.49	7.12
16.74	11.15	12.82	15.43	59.77	29.08	14.31	NA	94.62	45.28	29.39	60.08	14.14	3.85
11.65	6.23	7.18	9.16	58.11	21.31	12.28	11.48	103.76	44.69	28.18	59.22	7.51	2.85
10.99	8.48	14.76	8.91	56.98	22.71	12.2	NA	100.94	46.21	28.13	57.1	6.53	4.34
10.98	9.11	10.85	11.17	57.86	28.52	12.81	9.4	107.9	47.51	27.99	61.08	6.23	3.33
12.59	8.43	10.29	13.09	60.77	35.84	15.35	9.34	98.06	49.54	30.5	60.58	5.47	4
19.13	10.18	10.22	15.67	62.74	26.41	12.68	12.79	106.57	56.31	33.66	NA	4.84	2.67
11.34	5.99	7.85	8	53.31	17.87	9.23	8.88	96.49	48.62	24.48	49.78	7.01	3.65
14.91	10.22	11.28	10.99	52.69	25.34	13.58	10.66	96.77	47.33	31.01	52.19	13.42	NA
11.48	10.23	11.17	12.09	56.95	25.64	13.68	12.96	96.69	43.63	30.29	54.28	13.04	5.79
15.1	7.59	NA	14.79	63.41	NA	NA	NA	103.73	48.86	36.28	NA	12.19	6.3
12.62	8.3	12.99	10.91	62.32	22.55	11.89	12.8	97.28	60.93	32.86	58.4	12.65	6.94
16.04	6.47	9.91	9.99	53.7	25.63	13.89	12.09	102.75	50.17	29.68	52.99	11.23	6.28
16.67	8.12	12.2	10.21	54.22	24.06	9.38	11.4	91.22	48.12	31.39	55.46	11.13	8.32
10.63	8.17	10.79	8.26	55.85	27.59	NA	8.43	92.71	47.46	27.1	47.72	13.31	6.64
22.45	14.51	32.77	48.65	35.02	30.13	17	14.62	124.36	184.96	15.3	2	13.16	13.11
22.65	14.51	41.11	35.6	31.82	NA	NA	NA	109.93	174.76	NA	2	NA	NA
NA	15.41	43.58	42.91	45.09	NA	12.51	14.48	143.99	206.87	NA	2	NA	NA
19.41	9.4	25.56	38.42	28.73	20.66	11.54	13.92	105.45	164.33	13.83	2	10.98	13.7
6.15	4.35	8.06	NA	119.3	55.07	9.27	10.92	109.76	92.61	57.9	72.81	3.13	7.73
9.56	5.45	5.07	75.25	113.81	56.04	12.72	NA	112.72	95.05	68.11	NA	2.47	10.29

8.51	9.53	6.07	72.88	108.17	56.36	9.1	6.62	110.47	98.83	NA	66.54	2.93	8.45
11.32	11.22	10.52	74.55	105.43	60.42	15.46	13.35	112.4	100.73	68.76	58.08	NA	8.71
6.76	5.93	7.14	66.08	104.87	NA	10.09	9.09	108.36	96.75	52.24	55.63	3.12	8.99
8.76	7.66	7.06	83.83	112.62	68.35	11.18	8.86	114.03	89.37	71.17	72.08	2.69	10.87
9.41	6.05	7.11	79.53	111.15	NA	11.04	9.04	116.03	89.64	66.47	61.04	3.37	12.02
7.42	6.78	10.76	86.04	114.51	67.06	11.86	8.45	126.74	100.39	74.43	65.79	2.33	9.66
9.55	5.86	6.3	87.86	111.32	62.86	13.38	8.6	118.1	90.23	67.63	59.23	1.88	9.55
9.97	9.36	9.94	82.91	121.66	68.51	12.36	8.1	128.48	101.39	79.4	65.23	3.88	11.54
13.75	6.05	14.26	117.41	100.94	NA	NA	NA	115.25	113.8	78.45	71.08	14	6.39
11.4	4.39	12.25	97.69	96.71	64.88	21.05	11.8	92.54	98.67	66	83.43	14	7.5
11.92	7.33	17.81	119.8	113.01	70.51	18.48	15.18	114.7	108.47	83.69	61.5	16.21	7.49
5.39	4.94	10.2	95.61	225.47	90.46	13.45	9.69	123.33	92.79	77.84	95.21	16.14	3.72
8.52	7.41	14.09	NA	231.59	NA	18.1	12.82	133.35	91.23	77.12	NA	17.12	5.13
28.91	11.46	16.98	174.58	191.68	109.96	21.61	14.86	204.73	241.84	24.55	40.76	NA	NA
31.71	11.38	18.78	198.34	238.26	147.22	12.74	11.02	201.33	234	37.55	40.39	14.56	4.27
34.98	7.38	13.47	173.47	205.66	100.04	16.49	14.02	174.21	223	35.25	35.21	NA	NA
22.3	9.46	12.66	NA	173.11	137.08	13.88	11.05	167.96	206	27.41	40.95	11	3
19.86	12.62	13.66	170.25	234.41	NA	14.14	17.16	183.99	227	38.26	NA	NA	NA
14.2	6.85	12.1	187.21	NA	124.03	14.66	15.16	172.87	233	36.05	37.84	NA	NA
8.38	9.35	11.52	85.26	106.12	131.97	52.09	33.76	114.47	60.13	75.89	52.39	2.36	5.64
7.83	7.35	14.26	76.87	100.71	NA	50.25	35.48	102.69	56.98	71.85	47.55	3.03	6.35
7.62	5.76	7.46	81.09	110.57	130.51	48.46	34.48	87.68	50.09	75.77	49.15	2.45	9.16
6.1	8.58	6.65	84.78	103.72	111.73	48.17	29.71	NA	44.22	73.4	49.32	3.13	5.74
5.52	6.26	4.11	74.37	92.49	123.9	45.4	33.11	90.96	53.25	66.3	45.25	3.61	9.04
5.8	6.81	7.43	71.88	101.03	123.89	44.64	31.28	88.33	53.34	72.28	48.97	1.1	11.12
5.16	7.03	6.3	72.29	95.8	120.65	39.72	31.38	98.88	49.91	70.92	50.54	NA	NA
6.11	4.62	4.78	80.66	101.94	117.31	46.2	32.35	97.44	63.79	61.03	48.68	1.37	7.92
4.36	3.91	3.87	73.09	93.98	111.68	46.71	30.12	91.16	55.16	58.78	44.86	1.39	7.48
7.04	4.31	4.99	62.58	91.4	112.85	41.11	25.13	97.25	51.33	65.34	47.08	1.25	6.69
7.4	2.96	8.98	78.37	97.29	124.31	38.18	25.68	110.7	55.51	69.58	46.2	NA	NA
7.03	4.59	6.92	75.19	91.65	117.14	36.75	26.41	89.78	58.81	64.69	48.49	1.73	9.07
6.69	3.86	7.17	69.51	88.92	116.81	50.32	29.1	102.07	52.52	63.82	48.59	1.95	8.8

6.49	4.45	5.45	72.02	101.17	116.73	42	25.27	96.17	65.89	63.89	49.23	0.96	7.97
6.1	5.74	5.91	71.18	105.55	118.72	42.87	33.54	99.7	59.2	65.6	49.38	1.62	8.79
8.12	4.88	4.53	75.66	95.22	113.81	40.58	28.1	106.16	53.42	70.59	45.88	1.95	7.05
6.08	6.17	6.86	84.64	NA	127.98	42.8	29.52	105.1	54.04	68.69	NA	1.6	9.12
8.57	4.8	8.04	70.47	93.1	112.64	46.67	27.99	97.73	57.09	61.13	42.61	0.89	7.63
6.31	3.6	5.75	65.31	83.63	124.06	43.77	25.06	95.8	46.64	65.44	45.75	1.5	10.49
8.03	5.69	7.03	73.68	99.78	123.45	53.44	25.06	86.52	48.96	66.22	48.94	NA	NA
7.83	4.55	5.44	63.9	78.03	114.16	44.36	21.82	94.22	44.01	64.71	46.08	2.55	8.78
5.68	2.58	6.41	78.96	84.08	113.77	44.28	21.79	90.44	43.31	68	43.24	1.72	9.65
5.22	3.65	2.82	66.33	85.87	113.65	41.77	27.6	91.17	51.36	61.29	45.64	2.01	8.7
5.37	5.22	5.15	70.83	93.3	110.28	46.6	25.9	97.76	52.62	70.19	47.17	2.29	9.21
5.98	3.45	3.86	66.18	88.96	123.36	37.94	24.23	93.84	43.92	61.13	46.8	2.01	12.25
9.03	5.98	4.06	66.38	89.82	105.86	39.7	29.21	94.44	50.98	61.88	42.51	1.4	11.22
5.37	3.13	5.04	69.95	95.45	112.08	46.39	27.67	97	47.55	64.11	48.55	0.98	8.56
4.54	4.39	5.97	85.27	118.27	133.29	45.54	26.34	104.43	59.86	78.79	49.29	2.17	8.55
5.58	6.59	7.43	69.32	96.36	102.36	38.09	27.74	92.39	63.94	62.93	40.28	2.13	6.87
5.79	7.86	8.72	71.68	91.32	103.85	38.1	23.72	96.19	92.15	70.71	44.29	3.07	6.73
4.77	5.44	11.61	8.73	14.73	12.24	14.91	10.12	NA	NA	17.62	NA	NA	NA
14.74	13.76	11.65	14.78	28.43	18.48	16.24	14.73	NA	NA	19.86	50.87	49.53	1.64
16.02	14.08	18.57	14.19	22.89	16.32	11.16	14.15	NA	NA	NA	44.04	38.75	3.53
12.48	8.71	10.69	16.27	17.49	23.42	NA	NA	NA	NA	23.27	46	38.07	1.53
8.77	3.69	8.66	16.5	19.04	12.5	10	8.73	NA	NA	45.7	54.87	4.72	7.05
9.43	6.33	10.86	10.26	16.33	15	11.03	7.08	NA	NA	46.15	51.3	3.63	9.83
5.81	10.51	6.83	17.6	16.97	13.62	6.01	9.34	NA	NA	39.59	55	2.5	7.5
25.97	16.02	32.22	47.46	55.2	29.41	30.9	27.9	91.65	54.28	NA	18.31	NA	NA
24.1	13.4	32.58	44.32	53.17	30.34	NA	32.09	95.63	53.82	25.19	15.51	4.64	7.89
15.73	14.81	30.27	43.71	42.07	29.04	23.65	23.4	86.79	45.03	17.78	16.52	3	8
22.29	17.63	26.71	55.65	51.55	28.37	23.3	21.17	101.39	45.87	23.35	16.43	2.51	7.42
21.54	17.65	28.65	48.32	63.03	33.61	24.72	25.46	95.2	60.2	23.84	15.41	4.1	9.41
28.95	19.42	21.46	48.16	49.39	24.11	26.25	22.16	98.17	63.47	26.89	18.17	3.23	6.11
23.58	17.2	31.05	42.24	50.59	34.02	27.89	22.54	92.84	50.93	26.58	16.48	3.22	7.35
26.95	18.52	30.41	47.48	55.05	30.41	26.69	28.15	97.63	59.11	26.53	NA	3.73	7.65

22.8	16.59	26.46	50.73	54.39	30.03	28.37	28.55	99.34	50.63	26.99	16.64	2.16	6.25
26.44	19.64	41.88	59.61	52.14	38.9	34.12	32.7	105.08	55.3	29.39	NA	3.33	9.15
